# Sustainable Extraction Technology of Fruit and Vegetable Residues as Novel Food Ingredients

**DOI:** 10.3390/foods14020331

**Published:** 2025-01-20

**Authors:** Shiqi Zheng, Zhoumei Huang, Li Dong, Daotong Li, Xiaosong Hu, Fang Chen, Chen Ma

**Affiliations:** National Engineering Research Center for Fruit and Vegetable Processing, Key Laboratory of Fruits and Vegetables Processing, College of Food Science and Nutritional Engineering, China Agricultural University, Beijing 100083, China; zshiqi977@163.com (S.Z.); 18771235761@163.com (Z.H.); li_dong127@163.com (L.D.); lidaotong@cau.edu.cn (D.L.); huxiaos@263.net (X.H.); chenfangch@sina.com (F.C.)

**Keywords:** fruit and vegetable residues, sustainable extraction, novel food ingredients

## Abstract

Background: Fruit and vegetable waste (FVW) is a global waste issue with environmental impacts. It contains valuable compounds such as polysaccharides, polyphenols, proteins, vitamins, pigments, and fatty acids, which can be extracted for food applications. This study aims to review sustainable extraction methods for FVW and its potential in the food industry. Methods: This paper provides an overview of the sources and sustainable methods of high value-added compounds extracted from FVW. Sustainable techniques, including supercritical fluid extraction and ultrasound-assisted extraction, are compared with traditional methods, for their efficiency in extracting high-value compounds from FVW while minimizing environmental impact. Discussions: Sustainable extraction of FVW compounds is sustainable and beneficial for novel food ingredients. However, challenges in scalability and cost need to be addressed for wider adoption in the food sector. Conclusions: Sustainable extraction techniques effectively extract phytochemicals from FVW, preserving bioactivity and reducing environmental load. These methods show promise for sustainable food ingredient development.

## 1. Introduction

Fruits and vegetables are important foods and nutritional sources for humans. They can be consumed directly or processed into other products as ingredients. The edible portion of fruits and vegetables varies considerably from one species to another, and the inedible portions, considered as residues, include roots, stems, leaves, peels, seeds, and pomace. For example, the inedible part of passion fruit is up to 45–50% [1], that of rambutan is 50–65% [2], and that of mangosteen is 60–75% [3]. Table 1 shows the proportion of the nonedible parts of some other fruits. In addition to the residues, fruit and vegetable by-products (FVBP) are generated during the processing, transportation, and storage of fruit and vegetable products. A broader term, fruit and vegetable waste (FVW), which refers to the indigestible portion that is discarded at various stages during processing, can be considered as a kind of loss instead of waste. Including picking, grading, transportation, storage, marketing, and processing, loss occurs throughout the supply chain, and up to 50% of fruits and vegetables are wasted before they even reach the consumption stage [4]. Due to the higher production of fruit and vegetable products in the recent 10 years, as well as the lack of appropriate processing and handling methods, significant losses and wastage can be seen [5]. According to a report by the Food and Agriculture Organization of the United Nations (FAO), about 1.3 billion tons of food produced globally each year is lost or wasted, and the figure is still rising. One of the Sustainable Development Goals (SDGs) is to reduce food waste by 50%, a key challenge for FAO in 2050. In order to mitigate the enduring adverse economic, social, and environmental outcomes of ongoing waste generation, the European Commission has embraced a framework for advancing a circular economy [6]. Waste is recognized as the raw material for the circular economy, which is based on the socially driven concept of a zero-waste economy [7]. The disposal of such waste is a major problem affecting the environment. This problem can be solved by valorizing fruit and vegetable waste to recover economically important chemicals, and further to create a more sustainable agricultural system. A lot of research works are ongoing to recover commercially important from fruit and vegetable waste [8]. Consequently, focusing on the rational treatment and reuse of FVW is an important way to realize a low-carbon economy and ecological cycle.

**Figure 1:**
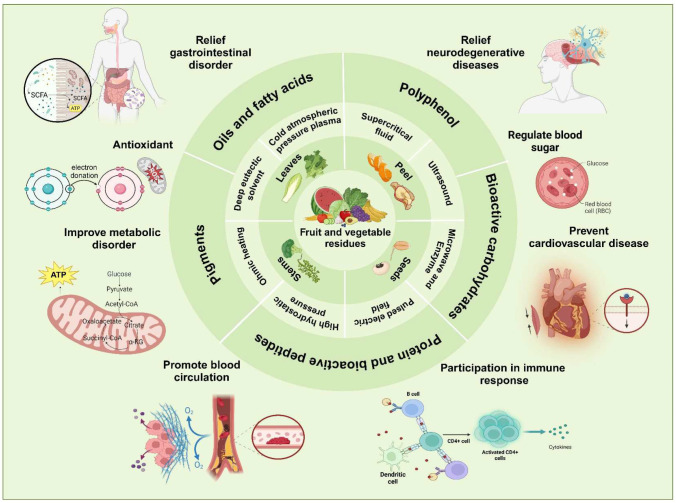
Graphical illustration of the functional component of fruit and vegetable residues.

FVW contains a lot of functional components and bioactive constituents such as alkaloids, amino acids, pigments, enzymes, polyphenols, proteins, tannins, saponins, vitamins, and terpenoids (Figure 1). Bioactive ingredients are the main components of biomass, which can produce favorable effects on human health. Numerous studies have been reported on the importance of polyphenols in the regulation of intestinal flora and improvement of cardiovascular diseases, insulin resistance, and systemic inflammation [9]. Plant tissues rich in dietary fiber have antioxidant and antidiabetic properties, thus aiming to prevent intestinal and metabolic diseases in humans [10]. Carotenoids act as potent antioxidants and natural food coloring agents, which can protect vision, heart, and skin health [11]. These bioactive compounds can be utilized in the food, cosmetic, and pharmaceutical industries as nutritional enhancers, antioxidants, bacteriostatic agents, or for other purposes [12]. Plants, bacterium, fungi, algae, and genetic engineering are the main sources of bioactive substances and the means of industrial production [13]. Given the growing demand and market potential for a wide range of bioactive compounds, it is important to develop alternative options. Therefore, the extraction of functional components from fruit and vegetable residues has significant prospects for development.

There have been many previous studies on the extraction and reprocessing of bioactive compounds in FVW, and the traditional extraction methods include: solid–liquid extraction, Soxhlet extraction, and liquid–liquid extraction [14]. However, these methods have obvious drawbacks, such as high solvent consumption, low extraction efficiency, limited extraction scale, risk of thermal degradation of thermally unstable components, and long extraction time [15]. This review categorizes the sources of functional factors and corresponding high value-added components in FVW, while focusing on emerging technologies from the source to the final product. In addition, possible avenues for the further development of extracts into products as novel food ingredients are explored, and this component to further enhance the value of the waste stream can be utilized. And finally, the main issues behind the development of laboratory methods into actual marketable products are discussed.

## 2. Composition of Fruit and Vegetable Residues

Fruit and vegetable residues are important sources of a neglected class of plant-derived compounds, the functional constituents of which vary widely depending on their source and structural site. The types of phytochemicals found in several common fruit and vegetable residues are listed in Table 2. Fruit and vegetable peels are vital sources of pigments, polyphenols, and enzymes. Banana peel contains large amounts of pectin, flavonoids, biogenic aminesa, and sterols, which suggests that it is a reliable source of functional polysaccharides for extraction [16]. Polyphenols in citrus peels exist in the form of polyphenol acids, mainly including caffeic acid, p-coumaric acid, ferulic acid, and erucic acid [17]. Additionally, onion skins have an abundant amount of anthocyanins and carotenoids [18]. Roots and leaves are rich in polyphenols, amino acids, dietary fiber, and chlorophyll [19]. The main functional components in grape stems are resveratrol and condensed tannins [20], while the seeds are abundant in lipids and fat-soluble substances, serving as an essential source of oil and pigment, and thus being of great value.

## 3. Functional Components of Fruit and Vegetable Residues

### 3.1. Polyphenol

Polyphenols are a kind of natural plant secondary metabolites with antioxidant properties, which can be involved in the treatment of differential human diseases. Based on their structural features, polyphenols can be categorized into benzoic and cinnamic acid derivative polyphenol acids and flavonoid and non-flavonoid polyphenols, with subgroups of flavonoids including flavanones, flavanols, anthocyanins, and isoflavones [36]. Polyphenols have obvious antioxidant properties and play an essential role in the prevention of several chronic diseases associated with oxidative stress, such as cancer, cardiovascular diseases, and neurodegenerative diseases [37]. Polyphenols have been shown to bind to immune cell receptors and can take part in modulating intestinal mucosal immune responses, allergic diseases, and antitumor immunity [38]. In addition, relevant beneficial effects can be seen in the gastrointestinal digestive system. Polyphenols are effective in attenuating cellular damage induced by oxidative stress and reducing damage to the structural morphology of the digestive mucosa [39]. Another important factor deserving attention is that polyphenols are polymerized to different degrees, which affects their bioavailability and metabolite production [40]. Dietary polyphenols are usually in a highly polymerized state, mainly in the form of esters, glycosides, or polymers, which cannot be absorbed by intestines in their natural form. They reach the colon directly and interact with intestinal microorganisms. In the colon, enzymes break them into absorbable small molecules for further action [41].

Current common sources of industrial polyphenol extraction are plants. Tea polyphenols, mainly catechins, are extracted from tea leaves [18]; proanthocyanidins are extracted from grape seeds and are commonly used in the extraction of high-purity polyphenol products [42]; hydroxytyrosol, extracted from olive leaves and olive fruits, is widely used in nutraceutical and cosmetic products; phenolic acids and oatmeal amides are extracted from the bran of oats, barley, and other cereals [43].

A number of FVW contains more polyphenols than the pulp fraction. In some fruit species, polyphenols in the peel account for above 40% of the total content in the fruit. For example, mango peel has a higher polyphenol content than mango pulp at all growth stages of the fruit [44]. And a polyphenol release of 63–82% from apple flesh and 42–58% from the peel was estimated [45]. The pericarp of kiwifruit was found to be more abundant in polyphenols and flavonoids than the flesh, with contents of 12.8 mg/g and 2.7 mg/g, respectively [46]. Berry hulls and pomace contain high levels of anthocyanins, which are responsible for their high total polyphenol content and antioxidant activity. For example, blackberry, blueberry, and Jaboticaba peels; strawberry hulls [47]; and raspberry pomace [48], among other berry peels or residues were able to extract adequate amounts of anthocyanins. The polyphenol content varies from one source to another. Tremocoldi [49] determined the polyphenol content in the peel extracts of two species of avocado and detected that the total polyphenol content in the peel (63.5 and 120.3 mg/g, respectively) was higher than that in the seed extracts (57.3 and 59.2 mg/g, respectively). Makori [23] extracted the potato peels from four potato varieties and the extracted polyphenol contents ranged from 360.61 to 157.20 mg CAE/100 g DW, which were all higher than the corresponding polyphenol contents in the pulp of the four varieties. Phumat [50] explored the functional properties of the extract from the fruit peel of *Benincasa hispida*, a food waste, with the ethanol extract (95%) demonstrating optimal efficacy in anti-aging and antioxidant activities.

### 3.2. Bioactive Carbohydrates

Polysaccharides, the most widely studied kind of metabolites other than polyphenols, constitute a diverse family of natural polymers consisting of aldoses or ketoses linked with glycosidic bonds. Polysaccharides in plants are usually structural components of plant cell walls and possess a variety of biological activities such as antimicrobial, blood glucose regulation, blood pressure lowering, blood circulation enhancement, immunity regulation, antitumor [51], hypoglycemic [52], gastrointestinal [53], and neuroprotective effects. Some of the polysaccharides can be absorbed and utilized to perform their respective functions. For example, dietary fiber (DF), a kind of undigestible polysaccharide, is the most abundant carbohydrate in plants, including hemicellulose, cellulose, and pectin. The prebiotic role of DF is well established through the formation of short chain fatty acids (SCFAs) [54]. Among them, pectin is a complex heteropolysaccharide, which is widely recognized as a valuable soluble dietary fiber component. And it plays an important role in human diet and health by lowering circulating cholesterol levels, decreasing postprandial glucose response, and increasing satiety, thereby reducing energy intake [55].

Wheat bran, bamboo, and sugar cane are important sources of industrially produced dietary fiber [56]. The main sources of pectin industrial production are citrus, lemon, and grapefruit peels [57]. Polysaccharides are commonly associated with plant epidermis and stems, and FVW has been shown to be a major source of natural plant polysaccharides. *Aloe vera* is a common plant rich in phytopolysaccharides. Minjares-Fuentes [58] studied the composition of polysaccharides in the non-edible parts of *Aloe vera*, which consisted of two main groups, an acetylated mannose-rich polymer that functions as the storage polysaccharide, and a galacturonic acid-rich polymer as the main component comprising the cell walls of the parenchymatous tissue. Polysaccharides in legume residues have been extensively studied. Soybean polysaccharides (SPS) are a class of soluble polysaccharides derived from legume residues that consist mainly of monosaccharide components such as glucose, galactose, and arabinose [59]. Citrus fruits are considered to be a reliable source of DF, accounting for about 25.71–80.41% [60]. Seeds of various fruits are also rich in DF. Grzelak [61] reported that DF is the main component of skimmed strawberry seeds, accounting for 700 g/kg DW. Apricot seeds contain about 27–35% of DF and grape seeds and date seeds account for 92.4% and 40%, respectively. Cranberry seeds contain 51.06% of total DF [62]. In addition, plant peels and stems are rich in pectin. Petkowicz and Williams [63] isolated pectin from broccoli stems by ethanol extraction with galacturonic acid (GalA) and degree of esterification (DE) content within the range set by the FAO and EU for food applications.

Polysaccharides extracted from FVW are shown to be used effectively in the medical and health field. Olennikov [64] elucidated the waste generated by the blueberry processing industry and its potential functionalities. The results of in vitro and in vivo experiments revealed that the polysaccharides in blueberries can effectively degrade the lipid profiles of hamsters fed a high-fat diet, ultimately validating the notion that food processing waste can be employed in the pharmaceutical industry for the production of hypolipidemic and antioxidant compounds. Zhu [65] extracted polyphenols and polysaccharides from corn silk, and identified the polysaccharides in corn silk as the effective hemostatic component, and flavonoids and saponins as the anticoagulant active components. And polysaccharides from various sources of corn silk confirmed their hemostatic effects.

### 3.3. Protein and Bioactive Peptides

FVW, especially seeds of tropical and subtropical fruits, is a considerable source of proteins and amino acids, and many specific amino acid sequences have been proved to have health effects [66]. In particular, bioactive peptides (BPs) have been a hot research topic in the last few years. BPs are composed of 2–20 amino acids linked by peptide bonds that are inactive in their parent protein sequence but can be hydrolyzed and released after enzymatic, chemical, and microbial action. They are known for their ability to inhibit protein interactions due to their small size and specificity [67]. BPs have antithrombotic, anticancer, antihypertensive, immunomodulatory, mineral binding, antimicrobial, and antioxidant properties. And they can prevent chronic diseases, particularly cardiovascular diseases and metabolic disorders, as well as having hypolipidemic and hypoglycemic effects [68].

Plant proteins have received a lot of attention in recent years as alternative proteins. The main sources of industrial production plant proteins are legumes such as soybeans, mung beans, and peas [69]. But the widespread use of soy protein also has some serious problems including protein digestibility and soy odor, and more proteins from plant waste are being incorporated into the food industry. Walnut residue is a by-product of walnut oil, which contains about 40–45% proteins of 18 amino acids, providing more than 95% of the antioxidant activity of walnuts [70]. Broccoli is known as a high-protein vegetable with a distinct amino acid profile and 42 kg/ton DW of total protein can be extracted from broccoli leaves [71]. Moreover, *Carica papaya* seeds with a protein content of 27.3–28.3% *w*/*w*, are the highest among subtropical fruit seeds [72].

### 3.4. Other Bioactive Substances

In addition to the major phytochemicals, vitamins, pigments, fatty acids, and other substances are also found in abundance in FVW which may have a variety of health effects. Carotenoids are naturally occurring plant pigments enriched in fruits and vegetables, and may reduce the risk of degenerative diseases such as cancer, cardiovascular disease, age-related macular degeneration, and cataracts [73].

Common sources of carotenoids are plants, microalgae, fungi, and metabolic engineering. Among them, algae possess some additional pathway for the synthesis of algae-specific carotenoids, capable of producing large amounts of carotenoids. Thus, it is the main source of carotenoids in the current food industry [74]. The methodology for the commercial production of carotenoids from marine microalgae, such as *Haematococcus*, *Dunaliella*, *Chlorella*, and *Spirulina*, has been standardized [13].

Liu et al. [21] noted that carotenoids, chlorophylls, and vitamins E and K in broccoli leaves were significantly more abundantly accumulated in leaves than in florets, suggesting that broccoli leaves may be a favorable source of vitamins and pigments. Lycopene is one of the most common carotenoids, and its main natural source is tomato [75]. Silva et al. [76] extracted 1446.6 μg/g lycopene from tomato processing waste. In addition to carotenoids, other natural phytochromes are also found in abundance in FVW. Beet is the richest source of betaine, a red pigment with a wide range of health benefits, which can be used as a natural colorant and has the advantage of being stable at a low pH [77].

Oils and fatty acids are relatively common in seeds, with residues of citrus fruit seeds, mango seeds, apricot seeds, pomegranate seeds, and tomato seeds containing significant amounts of oils, about 10–50%. Unsaturated acids (mainly linoleic acid) predominate in papaya seeds and have a very high omega-6/omega-3 ratio, and linoleic acid promotes the production of ceramides, which help maintain the skin barrier [78]. Mango seeds contains 44–48% saturated fatty acids (mainly stearic acid) and 52–56% unsaturated fatty acids (mainly oleic acid), which can serve as a healthy alternative to cocoa butter [79]. Pomegranate seed oil is a rich source of pomegranate acid, which belongs to a class of conjugated omega-5 fatty acids that can mitigate breast cancer [78].

## 4. Applications of Sustainable Extraction Technology in Fruits and Vegetables

### 4.1. Supercritical Fluid Extraction (SPE)

Supercritical fluid technology is a method of extracting nonpolar compounds (mainly carotenoids and lipids) by utilizing temperature treatments between the boiling point and the critical point of the fluid to keep it in the liquid state, thus changing its temperature-dependent dielectric constant [80]. Depending on the polarity of its extracts, SPE can utilize water, CO_2_, and propane as solvents, which can effectively reduce the amount of organic matter. Thus, SPE is considered a green and economical technique and an effective method for extracting plant functional substances under mild operating conditions.

The extraction of polyphenols, flavonoids, and carotenoids from plants using SPE is a common technique. Table 3 demonstrates the extraction efficiencies under a variety of parameters. Kupnik et al. [81] analyzed the pomegranate peel extract obtained under SPE at 20 MPa and found that the highest polyphenol content (11,561.84 μg/g) was mainly ellagic acid. Lachos-Perez et al. [82] stated that the extraction of total phenols from orange peels using SPE (10 MPa and 110–150 °C) was a reliable replacement, higher than conventional solvent extraction methods (31.70 ± 1.46 > 7.75 ± 0.99 mg AG/g DOP). Madhumeena et al. [83] investigated the SPE method for the extraction of polyphenols from pineapple wastes, followed by the use of high-performance liquid chromatography to quantify the total phenol content (2.365 mg/g GAE) and ferulic acid (0.7697/100 g). Lau et al. [84] extracted lutein (3.81 ± 0.02, mg/kg DW) and carotenoids (177.29 ± 4.35, mg/kg DW) from corn kernels using subcritical fluids, and found that the lutein level was twice of the conventional extraction, and the carotenoid level was three times of the conventional extraction.

Romano et al. [143] used supercritical CO_2_ to extract bioactive compounds from citrus peels and observed that the best yields were obtained when 20% ethanol (20 MPa, 20 °C) and supercritical CO_2_ (30 MPa, 60 °C) were used as co-solvents for the extraction and the extracts contained the highest amount of naringin. And results showed an extraction dose of 35.26, 44.05, and 19.86 mg/g in orange, tangerine, and lemon peel extracts, respectively. It was proven that the use of supercritical CO_2_ and ethanol mixtures as solvents improves the extraction efficiency and uses fewer organic solvents. Similarly, the recovery of carotenoids from 15 kinds of carotenoid-rich FVW using liquid CO_2_ at a flow rate of 15 g/min, temperature of 59 °C, pressure of 350 bar, and 15.5% ethanol as a co-solvent showed that most samples recovered more than 90% *w*/*w* of total carotenoids. And the compounds with the highest extraction rate were β-carotene (80–100% *w*/*w*) [130]. Pellicano et al. [131] extracted carotenoid from tomato peels (550 bar, 80 min), obtaining 79% of the maximum oil yield. The resulting extract primarily contained lycopene (0.86 mg/100 g) and β-carotene (1.5 mg/100 g).

Most of the research on SPE has been focused on the laboratory level and less on industrialization. The complexity of SPE equipment, high investment and operating costs, as well as the use of high pressure are challenges to be overcome for the implementation of SPE at the industrial level. Due to low polarity of CO_2_, the extraction is limited to most non-polar compounds, not applicable to the extraction of sugar. It is difficult to realize the large-scale production of plant polysaccharides. To overcome this limitation, a low toxicity and low dose of chemical modifiers can be added to enhance the polarity, such as ethanol, methanol, water, and acetone [147].

### 4.2. Ultrasound-Assisted Extraction (UAE)

Ultrasound is a special type of sound wave ranging from 20 kHz to 100 MHz that is capable of generating high-frequency ultrasonic energy by utilizing the cavitation process [148]. Ultrasound-assisted extraction relies heavily on high-frequency energy, which results in the rapid disruption of the cell wall to accelerate heat and mass transfer, thus it is effective in reducing mucus retention of the target constituents, resulting in high extraction rates [149]. Ultrasound has been used to extract molecules and a variety of biological materials including polysaccharides, essential oils, proteins, peptides, dyes, pigments, and bioactive compounds [150].

Corbin et al. [151] used alkaline hydrolysis of flaxseed using 0.2 N sodium hydroxide followed by extraction at 25 °C and a 30 kHz ultrasound frequency for 60 min to obtain the maximum amount of hydroxycinnamic acid glucosides and flavonols. Wani and Uppalur [107] optimized the process parameters for the extraction of functional substances from papaya leaves by UAE. It was found that the extraction temperature of 62.84 °C, 19.98 min ultrasonication time, and 0.2 g/mL sampling amount led to the highest yield of total phenols (149.12 mg GAE/g), which was six times higher than the conventional hot water extraction (28.61 mg GAE/g). Similarly, Iftikhar et al. [109] optimized the UAE conditions for polyphenols from rye bran using response surface methodology, and it was found that the highest extraction efficiency of total phenols (245.74 mg GAE/100 g DW) was achieved at 29 min, 66 °C, and solid–solvent ratio of 1:45 g/mL. UAE led to a significant increase in the extraction of total phenolic and flavonoid contents compared with the conventional extraction method. Khan et al. [142] noticed that the extraction of flavonoids from orange peel using ethanol as a solvent (naringenin: 70.3 mg and hesperidin: 205.2 mg/100 g FW) under optimal conditions of 40 °C, 150 W and a ratio of 4:1 (ethanol–water). Mauro et al. [111] extracted anthocyanins from eggplant peels using ultrasound-assisted and methanol containing 1% TFA, with the highest extraction concentration of 1461 mg/kg DM.

Wang et al. [152] utilized ultrasound to extract polysaccharides from *Rosa canina* peel and obtained the highest yield of 5.16% ± 1.81% when the extraction temperature was 70.0 °C, extraction time was 66 min, water-to-raw material ratio was 13 mL/g and ultrasound power was 230 W. Similarly, the extraction rate of mung bean peel polysaccharide was 2.55% under the conditions of a solid–liquid ratio of 1:40, temperature of 77 °C, ultrasonic power of 216 W, and time of 47 min. An in vitro study revealed that the polysaccharide obtained from the extraction had a significant scavenging effect on hydroxyl radicals and enhanced the anti-lipid peroxidation capacity [89]. Phomurugan et al. [153] revealed that UAE resulted in a higher yield (8.9%) of pectin from sunflower heads. The same results can be seen in the extraction of pectin from other fruit peels. Guandalini et al. [154] used 50% ethanol and UAE to obtain pectin from phenolic extraction residues and rehydrated mango peels, and the UAE increased yields by 53% (from 5.6% to 8.6%) when compared to the conventional extraction method. Freitas de Oliveira et al. [155] optimized the process parameters for the UAE of pectin from passion fruit peel and showed that ultrasound power intensity and temperature of 664 W/cm^2^ and 85 °C, respectively, resulted in the highest yield (12.67%) but the smallest GalA content and DE value. The extraction rate was increased by 1.6 times during UAE compared to conventional extraction.

The low selectivity of UAE leads to complex purification requirements; high power affects physical effects and equilibrium and generates excessive heat, and power decreases with time and is difficult to distribute uniformly in the vessel. Therefore, it is not easy to apply this technology on a pilot or industrial scale. Cavitation effects are more pronounced in the area around the ultrasound probe, so the installation of the ultrasound probe and the stirrer should be well designed in large industrial chambers. More in-depth probe designs and parameter optimization make it possible to further expand the industrialization of UAE.

### 4.3. Microwave-Assisted Extraction (MAE)

Microwave-assisted extraction is a method of extracting phytochemicals using microwave energies from 300 MHz to 300 GHz [156]. It is based on the direct transfer of microwave energy to the material through the interaction of the molecules with the electromagnetic field and thus the direct fragment of the plant cells, and further increases the efficiency of separation of the functional components from the cells [157].

MAE was used to extract polyphenols from pomegranate peel with the highest extraction efficiency of 376 mg GAE/g DW [158]. A similar study had compared the efficacy of novel non-thermal methods such as ultrasonic- and microwave-assisted extraction with the conventional maceration process for the extraction of polyphenols from olive leaves. And MAE resulted in the highest extraction yield and shorter extraction time [159]. MAE was used to extract polyphenols from eggplant peel waste. It was found that higher microwave power and lower ethanol concentration and liquid–solid ratio resulted in higher extraction rates and an increase in total anthocyanin, phenolic, and flavonoid contents. It was also revealed that the max predicted extraction rate (3.27%) was obtained when the power, extraction time, liquid–solid ratio, ethanol concentration, and solvent pH were 269.82 W, 7.98 min, 5.01 mL/g, 73.49%, and 3.06%, respectively [160]. Rodríguez García et al. [115] optimized the MAE parameters for the extraction of polyphenols from broccoli, and the optimal MAE conditions for broccoli leaves were 80% methanol, 10 min, and 73.27 °C, while the highest total phenol content of broccoli leaves was 1940.35 ± 0.794 μg GAE/g DW.

Hesperidin was isolated from citrus peels with MAE, and 70% aqueous ethanol solution was used as the extraction solvent, which resulted in a max extraction efficiency of 86.8% (47.7 mg/g), which was superior to that of the conventional extraction method (13.1 mg/g). The microwave power used in the process was 1 kW at 2.45 GHz at 140 °C for 8 min [145]. Ho et al. [161] utilized ethyl acetate as a solvent for the extraction of lycopene (13.592 mg/100 g) from tomato peels with the assistance of microwaves (ratio 1:20 *w*/*v*; 400 watts; 60 s) and found that it significantly improved the recovery of all-trans lycopene.

In addition, cell wall fragmentation is more favorable for the extraction of macromolecules such as polysaccharides and proteins from FVW. Pectin was extracted from banana peels using 96% ethanol, and it was found that when the optimal extraction conditions were 60 °C and 102 min, with a liquid–solid ratio of 40% (*v*/*w*) and pH of 2.7, the maximum yields of pectin and DE were 14.34% and 63.58%, respectively [90]. Similar results were verified in protein extraction. Barrios et al. [96] used MAE to extract proteins from beer residue, coffee residue, and collard stems, with maximum yields of 93.7%, 60.3%, and 95.4%, respectively. MAE significantly increased the dry matter and crude protein content of broccoli leaves up to 34.40 m/m% [98].

At the industrialized level, instruments and equipment for MAE are more expensive and more difficult to operate than some of the emerging technologies [162]. In addition, the need for additional clean-up steps, restriction of polar solvent application, and thermal effects due to microwaves that may lead to decomposition of thermosensitive and oxygen-sensitive compounds are major limitations to the further commercialization of MAE. Other MAE methods such as nitrogen-protected, ultrasonic, high-pressure, and vacuum MAE, which are rarely used for phytochemical extraction, should be investigated in the future and are expected to serve as a breakthrough point for the industrial application of MAE [163]. In view of the obvious advantages of MAE, the combination of MAE with other extraction techniques should be vigorously optimized in order to improve selectivity.

### 4.4. Enzyme-Assisted Extraction (EAE)

Enzyme-assisted extraction is dependent on operating conditions and environmental factors. Under suitable pH and temperature conditions, its active site is able to bind to the plant cell wall, leading to a change in the shape of the enzyme to maximize the interaction. In this interaction, breaks in the cell wall bond are produced, which release the active ingredient [164]. In the EAE process, a variety of carbohydrate hydrolases such as cellulase, trypsin, xylanase, amylase, papain, pectinase, and hemicellulase are widely used [165].

The most common method for obtaining bioactive peptides is enzymatic hydrolysis because of its rapidity and specificity and the possibility of generating peptides of a specific reproducible molecular weight and composition [166]. In addition, it is possible to extract functional constituents with a specific efficacy due to the different molecular weights and compositions of the peptides. When amaranth seed hydrolysates are obtained by trypsin or alkaline enzyme digestion, the products have a strong inhibitory effect on angiotensin I-converting enzyme, thus having the ability to lower blood pressure [167]. Similarly, a peptide was obtained from apricot waste using alcalase by García et al. [168]. Conversely, a very high antioxidant activity was observed for the amaranth peptides produced by combining alcalase and flavourzyme in the hydrolysis process [169].

Enzymatic hydrolysis has also been widely used in the extraction of polyphenols and polysaccharides, especially as a pretreatment step, which can greatly improve extraction efficiency. Enzymes were used to extract polyphenols and polysaccharides from mangosteen peels. At the optimal conditions, the addition of enzymes generated phenolics of 104–111 mg (GAE/g DW) and much higher polysaccharides (32.86%) [170]. Enzymatic hydrolysis was used to extract lycopene from tomato peels. An improvement in lycopene concentration (10% *w*/*w*, DW) was obtained by a second enzymatic treatment. Moreover, a second enzymatic treatment was conducted using a protease cocktail, which increased lycopene content 20–30 times over that of untreated tomato peels [135]. Babbar et al. [91] reported that in the EAE of berry pomace, pumpkin pulp, and beet pulp extraction, the highest extraction efficiencies of galactose polysaccharide were 85.9%, 82.2%, and 89.8%, respectively, under suitable conditions.

For EAE technology, a great deal of research has been focused on the laboratory level. The feasibility of EAE application on an industrial scale is low because the enzyme is severely limited by environmental conditions [171]. The use of EAE as a pretreatment can be explored in conjunction with other extraction techniques, thereby increasing selectivity and decreasing isolation difficulty.

### 4.5. Pulsed Electric Field-Assisted Extraction (PEFAE)

Pulsed electric fields are capable of utilizing an electric field energy of moderate intensity (0.5–10 kV/cm) and relatively low energy (1–20 kJ/kg) applied in the form of multiple repetitions of short-duration voltage pulses (usually from a few microseconds to 1 millisecond) [172]. The principle is to enhance the electroporation effect of electric field plant tissue pairs to produce a mass transfer phenomenon, where the electric potential passes through the cell membrane and separates molecules based on their charge, creating pores in the membrane and thereby disrupting the plant cells and increasing the permeability of functional factors. This provides help for cellular chemical extraction.

Frontuto et al. [117] reported that pretreatment with the PEF at 1 kV/cm and 5 kJ/kg was sufficient to achieve a high level of potato pericarp tissue permeability, which resulted in the improved recovery of phenolic compounds during conventional extraction, with a total phenol content of 1180 mg GAE/kg FW. With conditions of 50% ethanol and E = 1.2 kV/cm, the electric field was proven to have the strength to extract the total phenolics in a cocoa bean shell and coffee silver skin and quantify the main methylxanthines (theobromine and caffeine) and phenolic compounds (epigallocatechin and 5-caffeoylquinic acid) present in the samples. The results showed that the PEF parameters for each matrix increased the extractability of bioactive compounds in the samples by 20% and 21.3%, respectively, compared with conventional extraction [119].

Several similar studies suggest that the PEF was most significant in the extraction of anthocyanins. Maza et al. used a PEF to extract polyphenols from grape skins (E = 4 kV/cm and U = 4 kJ/kg) and found that the PEF significantly reduced (25–37%) maceration time and considerably increased color intensity, anthocyanins, and total phenolic content [173]. PEF markedly increased (27%) the yield of total curcumin in turmeric with a condition of E = 2 kV/cm and U = 3.5 kJ/kg. And it was found that the yield further increased with higher specific energy input (14 kJ/kg) [174]. Luengo et al. [133] investigated the effect of pulsed electric fields of different intensities (3–7 kV/cm and 0–300 μs) on the extraction of carotenoids from tomato peel, and the PEF treatment of 5 kV/cm increased the carotenoid extraction rate by 39%. They further investigated the use of the PEF for betaine extraction from beet and reported that the highest betaine extraction of 775 μg/g was obtained at a 75 μs 4 kV/cm pulse treatment [175].

Yu et al. [100] investigated the PEF of proteins from oilseed rape stems and leaves, which was mainly dependent on the electric field strength of the plant and the growth stage. Specific parameters leading to tissue damage were observed at 800 V/cm and up to 80% protein extraction was achieved at 20 kV/cm.

Limitations of PEFAE include a lower effectiveness due to gas bubbles, reversibility due to membrane alteration, and the efficiency of the method being dependent on the electrode gap and electric field strength. Some studies have shown that applying the electric field at room temperature is not sufficient, so the temperature can be increased by using pulsed ohmic heating through ionic motion in a series [176]. The results obtained in the pilot unit are very promising for scale-ups, and it appears that up to 2500 tons/day of sugar beet and sugar cane flowers can be processed using current technology [177].

### 4.6. High Hydrostatic Pressure-Assisted Extraction (HHPAE)

High hydrostatic pressure technology utilizes pressure, typically operating at pressures between 100 and 1000 MPa, to alter the chemical bonding of the cellular structure, increasing mass transfer rates and cell permeability, allowing solvent diffusion, and increasing the diffusion of secondary metabolites in response to phase transitions [93]. This technology was initially used in the food sector to inactivate microorganisms and enhance food shelf-life [178]. In addition to food preservation, HHP has been shown to have the ability to increase the extraction efficiency of bioactive compounds and reduce processing time. Pressurized foods treated with HHP have shown higher levels of functional compounds such as phenols [179].

In a study on cactus leaves, it was demonstrated that the application of HHP at 100 MPa for 5 min helped to release phenolics attached to the cell wall and betaine in the cell vesicles through cell wall rupture modification [180]. Morata et al. [181] extracted anthocyanins from grape pomace using HHP, and the results showed that HHP treatments of 10 min at 200, 400, or 550 MPa did not differ in the amount of anthocyanins extracted, suggesting that the mechanical effect of HHP in anthocyanin extraction was already maximized at the lowest pressure (200 MPa). In contrast to the findings of Morata, Okur et al. [121] conducted several parallel experiments on different techniques for polyphenol extraction from tart cherry pomace. It was found that when the HHP treatment pressure was increased from 400 MPa to 500 MPa, the amount of extracted phenolics rose from 39.5% to 61%, with the final result of 227.51 ± 1.28 mg GAE/100 g FW.

HHP is also widely used in pectin extraction. The yields of pectin from Satsuma mandarin peel using two different solvents (citric acid and hydrochloric acid) ranged from 15.34% to 18.99% under a HHP treatment condition of 500 MPa for 10 min, and the results proved that using citric acid as an extractant at high hydrostatic pressure significantly increased the yield of pectin [92]. Different conditions (250, 350, and 450 MPa at 40 °C for 5 min with/without acid) of HHPE were investigated in the extraction of beet pulp pectin and compared with conventional pectin extraction; the extraction rate of HHPE doubled (12.23 ± 0.13%) [93].

Solvent selection and pressure conditions are important factors affecting extraction efficiency. Lara et al. [136] reported a process technology for the preparation of carotenoids from papaya peels using HHPAE with soybean oil as solvent. The results showed that under the optimum conditions of 400 MPa, 40.5 °C, and 5 min, a yield of carotenoids of 38.0 μg/g DW was produced. And they analyzed the composition of carotenoids in the extract, which consisted mainly of lycopene, carotenoids, cryptoxanthin, and lutein esters. Similar results were obtained in a study on papaya seeds, which noted that using a medium concentration of ethanol at 500 Mpa enabled the achievement of high antioxidant extraction efficiencies in a short period of time (6.49 ± 0.06%), and when the pressurization time increased from 5 min to 15 min, the extraction rate increased from 9.8% to 20.4% [138].

The advantage of HPE is the possibility of mixing different solvents with different polarities, which allows for the extraction of different compounds as well as the control of the amount of impurities present in the final extract [182]. It is important to ensure process uniformity and homogeneous temperature distribution during HHP processing. Large HHP food processing plants are ideal for the food industry and there is already industrial use of HHP sterilization, so the industrial application of HHP green extraction technology can be achieved after further parameter optimization.

### 4.7. Ohmic Heating-Assisted Extraction (OHAE)

In contrast to pulsed electric fields or high-voltage electrical discharge, the key distinction of ohmic heating-assisted extraction lies in the thermal properties, which allow for a wide range of waveforms and frequencies, typically sinusoidal, spanning from Hz to kHz, as well as the presence of alternating medium to low electric fields, potentially below 0.1 kV/cm [183]. Ohmic heating, in comparison with other thermal processes, is a uniform, rapid, and efficient electrical heating method. Its principle involves the generation of heat due to the resistance of the medium and the object itself when an alternating current passes through the object. Due to the superior electrode–liquid contact, ohmic heating facilitates uniform heat distribution [184]. In addition, because of the effect of the electric field, ohmic heating has additional non-thermal effects, such as electroporation, thus having a smaller impact on the structure, nutrition, or sensory characteristics of food compared with traditional thermal processing technologies, and it can achieve higher extraction efficiency.

Pectin extraction is an important application scenario for OHAE. Çilingir et al. [95] optimized the extraction process of lemon peel pectin using OHAE, and it was determined that under a voltage gradient of 11 V/cm, a solid–liquid ratio of 1:60 g/mL, and a retention time of 120 min, the highest extraction efficiency was achieved with a pectin yield of 18.51%. Pectin was extracted from orange waste using OHAE, and a study showed that temperature is the most important factor. The best extraction conditions for high-quality ohmic heating (high DE and galacturonic acid) pectin are a voltage gradient of 15 V/cm, a temperature of 90 °C, and a duration of 30 min [185]. In addition, OHAE also involves the extraction of small molecules such as polyphenols, betaine, and anthocyanins. El Darra et al. [186] extracted polyphenols from red grape pomace and revealed that the extraction rate of the grape pomace pretreated with OH (400 V/cm) increased by 36% compared to that of traditional methods. OHAE is also an effective process for extracting polyphenols from tomato by-products, with a recovery rate 58% higher than that of the control sample. For lipophilic compounds such as lycopene and β-carotene, OHAE allows their extraction from tomato by-products without the addition of organic solvents. However, their extraction yields are lower compared to those of traditional methods and require further optimization of the extraction process [187]. The concentration of phenolic compounds and antioxidant efficacy extracted from wheat bran using OHAE are the highest, and the obtained wheat bran extract can be used as a potential substitute for synthetic antioxidants [188]. Cabas and Icier [189] utilized OHAE to extract betacyanins and betaxanthins from sugar beets under varying solvent and electric field conditions. When using aqueous and ethanol extraction media, OHAE yielded higher amounts of lutein than traditional solvent extraction methods, and the optimal yields for betacyanins and betaxanthins were achieved at a voltage gradient of 17 V/cm and a frequency of 400 Hz. OHAE has also demonstrated its effectiveness in the extraction of anthocyanins from grape skin, with the total anthocyanin content in water extracts increasing significantly [183].

OHAE can be synergistically integrated with other extraction methods to substantially improve extraction yields. Nikoomanesh et al. [190] combined ohmic heating with ultrasonic and microwave techniques, and their study showed that extracts obtained using ohmic heating combined with ultrasound (US-OH) from burdock (*Arctium lappa* L.) exhibited significantly higher antioxidant activity than those obtained by other methods.

High implementation costs and lack of technical characterization of the method are among major drawbacks of OHAE, and a more significant challenge to adopting this technology on an industrial scale is the optimization and control of key parameters for each food material [191]. The choice of electrode material and design poses a major challenge to the durability of the equipment. Electrochemical reactions between the electrode and the food material can lead to electrode corrosion. Therefore, further optimization of electrode materials is a direction for development of OHAE.

### 4.8. Deep Eutectic Solvent-Assisted Extraction (DESAE)

Serving as alternatives to conventional organic solvents, sustainable solvents such as diethyl carbonate (DEC), 2-methyltetrahydrofuran (2-MeTHF), ionic liquids (IL), ethyl lactate, natural deep eutectic solvents (NADESs), and deep eutectic solvents (DESs) have been increasingly applied in the extraction of phytochemicals [192]. Among these, DES and NADES have emerged in recent years among the leading solvents for green extraction processes.

DESs or NADESs are mixtures obtained by blending a hydrogen bond acceptor such as quaternary ammonium salts or metal salts with simple hydrogen donors like alcohols, acids, amines, and amides [187]. Hydrogen bonding occurs between halide anions and the hydrogen donor components, creating a hydrogen bond network that enhances the system’s stability and results in distinctive physical properties. DESs can exhibit high viscosity, with melting points that are substantially lower than those of their individual constituents; even the melting points of certain NADESs can be below room temperature [193]. Moreover, DESs possesses a wide polarity spectrum, significant biological activity, and high solubility for a diverse array of compounds, which render them suitable for the extraction of phytochemicals.

A variety of DESs were developed for the extraction of polyphenols and anthocyanins from grape skins. A study revealed significant differences in extraction efficiency among DESs of varying solvent types and pH levels. A DES composed of choline chloride and fructose in a 1.9:1 ratio, with the addition of 30% water, achieved the optimal extraction rate of 100 mg/g dw and exhibited reduced cytotoxicity [123]. A NADES, which was formulated with a 1:1 ratio of choline chloride to malic acid, has been demonstrated to effectively extract anthocyanins from winery waste [194]. DESs demonstrate effective extraction of flavonoids; a study by Guo et al. revealed that a tailored DES composed of L-proline and glycerol outperformed methanol in extracting quercetin, kaempferol, and isorhamnetin from *Sophora flavescens* [140]. A DES solution prepared with a 1:6 ratio of choline chloride to acetylpropanoic acid was utilized to extract protein from the tips, bases, and sheaths of bamboo shoots, yielding 39.16 ± 1.22 mg/g DW, which is significantly higher than that of the conventional extraction using NaOH solution [102]. DES also exhibits high extraction efficiency for lipophilic plant compounds and effectively reduces the use of organic solvents. DESs and NADESs have emerged as promising substitutes for conventional solvents in the extraction of carotenoids and other natural compounds. Zhang et al. [195] employed switchable DESs in combination with phenolic antioxidants for the extraction of β-carotene from millet. Following five cycles of DES usage, the recovery rate of β-carotene from millet was maintained at 91.01 ± 0.96%, showcasing robust extraction and recovery performance.

It is important to study the recoverability and reusability of these emerging green solvents in order to enable industrial scale-ups. Strategies focused on improving extraction efficiency and solvent recovery or reuse usually involve high operating costs and energy consumption, and blending DESs with other solvents for extraction may be a possible solution that should be investigated in the future.

### 4.9. Cold Atmospheric Pressure Plasma-Assisted Extraction (CAPPAE)

Plasma is characterized as a quasi-neutral ionized gas state that predominantly consists of air, nitrogen, oxygen, helium, and argon, and is composed of ions, free electrons, atoms, and molecules in their ground or excited states, possessing a net neutral charge [196]. Plasmas are categorized into thermal and non-thermal plasmas based on whether they are in thermodynamic equilibrium and the extent of ionization. Non-thermal plasmas, commonly known as cold plasmas, are employed in the food processing sector due to their pronounced antibacterial efficacy against key foodborne pathogens such as *Escherichia coli*, *Salmonella typhimurium*, *Staphylococcus aureus*, and *Listeria monocytogenes* [197]. Furthermore, cold atmospheric pressure plasma (CAP) has been employed in a range of applications, including food drying, the processing of packaging materials, the functional modification of food matrices, and the removal of pesticide residues [198]. Research into the use of CAP for the extraction of phytochemicals has been advancing in recent years. It shows the capacity to disrupt cell wall structures and enhance surface hydrophilicity, thereby enhancing extraction efficiency.

Recent studies have demonstrated that the application of CAP as an auxiliary extraction method can significantly enhance the extraction efficiency and total phenolic content of polyphenols from FVW. Kashfi et al. [125] utilized 50 W of power for 20 min of plasma treatment on dried mint leaves, and their study revealed that while the CAP treatment induced a certain degree of color alteration, it considerably augmented the total phenolic content (292.53 mg GAE/g edw) and antioxidant potential. The combination of CAP pretreatment with cyclodextrins has been shown to elevate the total phenolic content and antioxidant potential of *Cornus officinalis* fruit residue extracts, with a concurrent improvement in the extraction and bioaccessibility of phenolic compounds during simulated in vitro digestion [199]. A study observed that cold plasma processing markedly increased the content of individual polyphenols extracted from rice bran (containing vanillin, ferulic acid, sinapic acid, and chlorogenic acid) and corn bran (comprising 4-hydroxybenzaldehyde, p-coumaric acid, sinapic acid, and ferulic acid), with the resulting polyphenols exhibiting enhanced in vitro digestibility, cell viability, and anti-inflammatory properties [127].

Input parameters such as power, voltage, processing duration, gas type, frequency, and gas flow rate play a pivotal role in the treatment process. Among these, working gas is a critical component, as it dictates the compounds that undergo activation. CAP under various gas conditions results in distinct effects on the total phenolic content of tomato residue. Samples treated with helium and nitrogen plasmas exhibited significantly enhanced phenolic extraction yields compared to that of the controls, with increases of 8.9% (1.025 ± 0.036 mg GAE/g) and 9.8% (1.033 ± 0.020 mg GAE/g), respectively, whereas argon plasma treatment did not demonstrate a significant improvement [200].

In addition, CAPPAE can be integrated with other extraction techniques as a pretreatment technique. For example, the integration of CAP with pulsed ultrasound-assisted extraction technology can be utilized to augment the extraction efficacy of bioactive compounds from *Limonia acidissima* [201]. In the enzyme-assisted extraction for the recovery of proteins from soybean meal, the protein extraction yield of the high-temperature soybean meal after CAP treatment reached 83.26 ± 0.89%, which is significantly higher than 70.03%, the yield achieved by sole enzymatic hydrolysis [202].

CAP has had some small-scale industrial applications, mainly focused on industrial sterilization. However, the application of CAP to the extraction of phytochemicals also comes with some challenges that hinder its applicability. Different generators and gasses have different effects on extraction efficiency and need to be further explored and selected [203]. Since the current instrument size is mainly used at a laboratory scale and may not be suitable for industrial operations that require continuous high-volume processing, upgrading and expanding the size of the instrument should be the main research direction in the future.

## 5. Fruit and Vegetable Residues as Novel Food Ingredients

The phenolic content present in FVW can be identified as a useful resource for food additives. Bioactive constituents such as carotenoids, phenolic acids, and flavonoids are commonly found in tomato peel, pumpkin seed peel and rind, papaya peel, and watermelon peel. And their presence in fruit wastes offers antioxidant properties that prolong the shelf-life of the product by delaying the formation of off-flavors and rancidity, and can be used as natural preservatives and coloring and indicating agents in food applications [204]. The incorporation of mango peel extract into chicken sausages may be an effective strategy because it increases thiol content and decreases base content, which helps prevent protein oxidation [110]. Lycopene from tomato skin can be used as a natural antioxidant for food products and can effectively reduce browning (browning index: 43.8) and extend shelf-life up to 9 days [106]. Some bioactive substances derived from plants are also used in the food industry as flavor enhancers. Vanillin from beet pulp and chlorogenic acid from grape waste can be used as flavoring agents to provide fruity flavors to food [108]. In addition, pineapple waste is also one of the sources of ferulic acid and a reliable alternative to food flavoring agents, which is considered a precursor of aroma compounds such as vanillic acid and vanillin [62]. Bioactive substances can also be used as additives to improve the physicochemical properties of foods. Tea polyphenol treatment could cause the aggregation of protein molecular chains in wheat gluten, thus improving gluten strength and noodle texture [124]. Pectin can be used as an emulsifier for dairy desserts and mayonnaise products, and as a fat substitute. A total of 35.58% pectin yield was obtained from novel source, i.e., *Citrus limetta* peel waste, by optimizing the ultrasound-assisted extraction process. Cookies prepared with the extract successfully replaced 30% of butter and had the most acceptable organoleptic score and physicochemical properties [205]. Cookies with 10 and 20% partially defatted tomato seed flour as a fat replacer were found to be more preferable and acceptable in terms of sensory characteristics and other quality characteristics [206]. Moreover, dietary fibers are also important additives in the food industry to increase the fiber content and to improve the polyphenols’ shelf-life, hydration, antioxidant properties, and stability of the products. Dietary fibers extracted from FVW have been successfully added to the preparation of bakery products such as cookies, cakes, breads, and muffins [207].

Bioactive substances extracted from FVW can also be used as nutrient enhancers with the potential to produce nutraceuticals and functional foods. Polyphenols extracted from green coffee beans as prebiotics have been incorporated into the formulations of probiotic yogurt successfully, providing high maintenance of the probiotic counts in the product [122]. The addition of grain extract to the biscuit may also serve as a nutritional booster. The addition of millets (pearl millet, finger millet) could be incorporated even up to the 40% level, indicating its suitability for obtaining biscuits with much higher nutrition and overall acceptability without considerably affecting the other quality parameters. Two-fold increases in protein, three-fold increases in dietary fiber, and a one-fold increase in mineral content were observed in multigrain biscuits [208]. A study chemically characterized polyphenolic compounds extracted from mango peels and seeds, then investigated the anti-obesity effect of the extracts on 3T3-L1 cellular ratings. Both peel and seed extracts were found to be promising anti-obesity natural compounds by down-regulating the key adipogenic transcription factor PPARγ and its downstream targets and inhibiting AMPK activation, thereby inhibiting acetyl coenzyme A-carboxylase and further reducing lipid accumulation and triacylglycerol content [209]. Aqueous-based extracts from the pea pods of active molecules were characterized, of which polyphenolic compounds were mainly represented by 5-caffeoylquinic acid, epicatechin, hesperidin, and catechin. And they were used to produce two different nutraceutical formulations (acid-resistant capsules and non-acid-resistant capsules). The extracts yielded an acid-resistant capsule formulation capable of retaining the active compounds in a simulated gastrointestinal digestive process [112]. Pinho et al. [113] concentrated their research efforts on the extraction of carotenoids from the skins of guarana berries and encapsulated them using spray drying to enhance their stability. The oatmeal paste was supplemented with a 1:2 concentrated ethanol extract-to-gum arabic ratio. The paste enriched with carotenoid particles displayed reduced viscosity, and in this encapsulated form, the carotenoids could be added to cooked foods at higher temperatures, thereby producing stable and beneficial products.

## 6. Safety of the Reuse of Fruit and Vegetable Residues

While current research on the reuse of FVW in the food industry should continue to be promoted, we must remain vigilant and open-minded to safety concerns. The potential presence of anti-nutritional factors and toxins should be the first concern in the evaluation and utilization of functional ingredients. The use of non-edible parts of plants may expose consumers to toxins produced by yeasts or fungi, such as aflatoxin B1. There are relevant studies evaluating the levels of heavy metals, oxalates, saponins, and cyanogenic glycosides in banana peels, concluding that their concentrations do not constitute a health hazard [210]. Another serious problem that may limit the reuse of many seeds (apricots, peaches, apples, cherries, plums, lemons, and almonds) is toxic hydrolyzed derivatives of bitter amygdalin (benzaldehyde and cyanide), which necessitates the establishment of a detoxification procedure prior to mandatory use [211]. In addition, the presence of pesticide residues should be assessed. Industrial and homemade washing procedures have been shown to significantly reduce levels of some pesticides, although they are not as effective for others [212]. Finally, potential allergens, such as peach skin and mango skin, should be evaluated. Due to the more specific origin and nature of these ingredients and the potential risk of sensitization, accurate and clear labeling may be challenging. Exaggeration, misuse, or improper narration of functional ingredients are also issues that need to be addressed. Therefore, there is a need to strengthen the review and regulation of labeling contents to ensure their truthfulness and accuracy. In terms of food regulations, novel food ingredients need to be authorized before they are allowed to be put on the market. Novel foods that have received authorization are specified in the Commission Implementing Regulation (EU) 2017/2470 of 20 December 2017 establishing the Union list of novel foods in accordance with the Regulation (EU) 2015/2283 of the European Parliament and of the Council on novel foods. The food included in the list may be placed on the market, if the conditions of use, specific labeling requirements, specifications, and other requirements indicated therein are complied with [213]. China’s latest Administrative Measures for the Safety Review of New Food Ingredients, revised in 2017, mentions ingredients isolated from animals, plants, and microorganisms as being under the regulation. Food ingredients that have no traditional consumption habits in China can be declared as new food ingredients after safety review. After review, they can be used in food production and management. The review process requires the issuance of a food safety assessment report and on-site verification of the production process. One of the problems to be faced is the clarification of the relevant standards to be implemented for new food ingredients, including safety requirements, quality specifications, and test methods, which is usually difficult. The introduction of more detailed standards should be the direction of the development of plant extracts as new food ingredients.

## 7. Limitations and Extensibility from the Laboratory to Industrial Production

As mentioned above, the industrialization of green extraction technologies creates some obstacles due to the disadvantages of different sustainable technologies in terms of instrument size, equipment selection, capital costs, energy consumption, and parameter optimization. The separation and purification of bioactive compounds can be very complex and may render the whole extraction process inefficient. Another obstacle to the effective utilization of sustainable extraction technologies is the difficulty of obtaining extracts that meet Good Manufacturing Practice (GMP) quality requirements, which hinders their use in final consumer products.

In response to these limitations, higher-quality parameter optimization models, such as artificial neural network modeling, will be applied to industrial design [214]. Enhanced understanding and the optimization of extraction conditions can improve extraction efficiency while reducing energy consumption and saving money. Pilot-scale studies are necessary to determine the best technology based on the cost-effectiveness of producing commercially viable extracts. Advances in nanocarrier and embedding technologies can better expand the use of extraction products in the food industry [215].

Sustainable extraction techniques in combination with each other can improve extraction efficiency and reduce costs. The most widely used green extraction technologies are MAE and UAE because of their maturity and multiple advantages. The synergistic approach of these two technologies with other novel extraction techniques can achieve high extraction efficiency and analyte selectivity. Short extraction times and moderate extraction temperatures can be achieved, helping to minimize possible thermal degradation problems.

## 8. Conclusions

**Figure 2:**
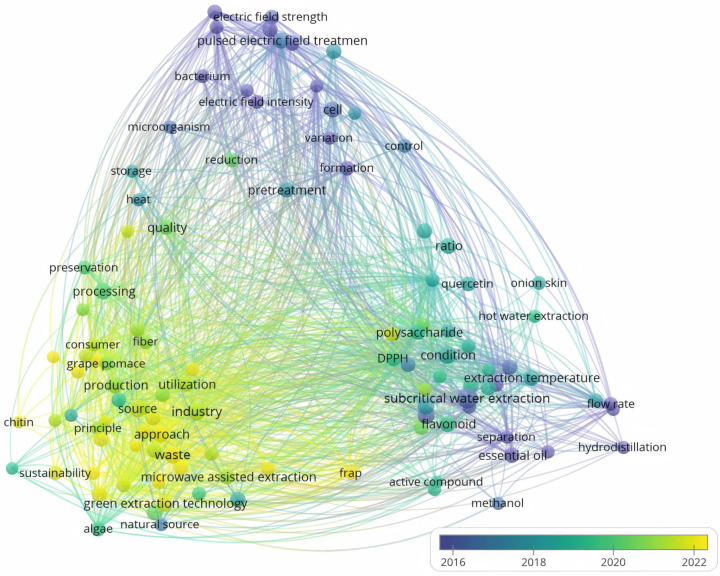
Extraction technique maps for phytochemicals from fruit and vegetable residues.

In recent years, emerging food ingredients have become a new trend in the food industry due to the surge in demand for health and environmental protection. More studies are focusing on the reuse of functional ingredients from FVW. However, for the wide variety of FVW, it is necessary to analyze the functional components contained in a specific and detailed manner. In addition, the optimization of the whole process, from raw material selection to extraction method application, should be the focus of attention. Many attempts have been made to produce bioactive compounds from FVW. Whereas novel extraction methods are able to extract active ingredients from fruit and vegetable wastes in a more economical, rapid, efficient and environmentally friendly manner. Till now, yield improvements have been shown in the use of ultrasound-, microwave-, supercritical fluid-, pulsed electric field-, or enzyme-assisted extraction. The survey of extraction techniques for phytochemicals in fruit and vegetable residues compiled by searching the relevant literature through VOSviewer at Web of Science is presented in Figure 2.

The application of FVW extracts as novel food ingredients needs to be further explored. The current industrial application of the extracts is mainly focused on food packaging, while few applications at the level of food ingredients have been reported. Therefore, the future fruit and vegetable waste reuse industry can be gathered towards the raw material part. At the application level, the safety of fruit and vegetable waste extracts as new food ingredients also needs to be evaluated to assess whether the process can be scaled up harmlessly. As mentioned above, only a few studies have explored this area so far, and the gaps in determining the toxicity of each extract in terms of antinutritional factors, allergens, and pesticide residues need to be filled.

In conclusion, the extraction of functional ingredients from fruit and vegetable wastes has the potential to generate new value-added products for food ingredients and production. In turn, this review may help to reduce food waste and waste management and promote a circular economy.

## Figures and Tables

**Table 1:** Major loss and waste of common fruits and vegetables.

Fruit and Vegetable	Typical Loss and Waste	Fruit and Vegetable	Typical Loss and Waste
Mangosteen	60–75%	Pea	40%
Durian	60–70%	Banana	35%
Rambutan	50–65%	Dragon fruit	30–45%
Pomegranate	50%	Tomato	20%
Oranges and tangerines	50%	Grape	20%
Passion fruit	45–50%	Potato	15%
Mango	45%	Pawpaw	10–20%

**Table 2:** Active components in several plant residues from different sources.

Source		Functional Component	Health Effect	
Peels	Banana	Carotenoids	Antioxidation, eye health, cardiovascular protection, anticancer	[16]
		Flavonoids		
		Biogenic amines	Neurotransmitter function, mood regulation, enhance metabolic vitality	
		Sterols	Cholesterol-lowering, prevention of cardiovascular system diseases	
		Terpenes	Anti-inflammatory properties, anticancer, neuroprotective	
	Apple	Chlorogenic acid	Antiviral, anti-diabetic, anti-inflammatory, anti-obesity	[21]
		Epicatechin	Diabetes and obesity management, improve cognition and cardiovascular function	
		Proanthocyanidin B2	Neuroprotective, anti-inflammatory, reduces risk of ischemic stroke	
		Phloretin	Immunosuppressive, cardiovascular and respiratory health	
		Quercetin	Antimicrobial action, anti-inflammatory	
	Citrus	Caffeic acid	Antioxidant, anti-inflammatory, anticarcinogenic, diabetes management, cardioprotective, neuroprotective	[17]
		Ferulic acid		
		Sinapic acid		
		Naringin	Promotes carbohydrate metabolism	
		Hesperidin	Lipid-lowering	
		Dietary fiber	Digestive health, cardiovascular protection, diabetes prevention	
	Pomegranate	Pomegranate	Antioxidant, anti-inflammatory, anticarcinogenic, cardiovascular health	[22]
		Granin		
		Ellagic acid	Neuroprotective, anti-inflammatory, antioxidant, anticarcinogenic	
		Gallic acid	Antimicrobial, antioxidant, anti-inflammatory,	
	Onion	Kaempferol	Cardioprotective, antioxidant, anti-inflammatory	[18]
		Luteolin	Cardioprotective, anti-inflammatory, antioxidant, anti-allergy, reduce uric acid	
		Quercetin	Antioxidant, reduce blood fat and pressure, enhance capillary resistance	
		Flavonols	Antioxidant, anti-inflammatory, anticancer, supports heart health	
		Anthocyanins		
		Proanthocyanidin B2	Neuroprotective, anti-inflammatory, reduces risk of ischemic stroke	
		Carotenoids	Antioxidant, supports eye health, immune support, anticarcinogenic	
	Potato	Chlorogenic acid	Antioxidant, anti-diabetic, anti-inflammatory, anti-obesity	[23]
		Flavonoids	Antioxidant, anti-inflammatory, cardiovascular health, neuroprotection	
		Anthocyanins		
		Carotenoids	Antioxidant, supports eye health, immune support, anticarcinogenic	
		Vitamin C	Immune system support, antioxidant, cardiovascular health	
	Mango	Fructose, xylose, and pectin	Contributes to gut health	[24]
		Maclurin derivatives	Antioxidant, anti-tyrosinase, anti-tumor, anti-proliferative	
		Gallates	Antioxidant, anticancer properties, anti-biosis	
		Protein	Essential for cell structure and function, crucial in every biological process	
		Gallotannins	Antioxidant, antibacterial, astringent properties	
		Flavonoids		
		Ellagic acid		
		Mangiferin	Treatment of chronic bronchitis, inhibit the central nervous system, anti-inflammatory, bacteriostatic, anti-herpes simplex virus	
Stems and roots	Ginger	Trans- ferulic acid	Antioxidant, antifertility, myorelaxant, and antispasmodic properties	[25]
		Epicatechin	Antioxidant, anti-inflammatory, neuroprotective, protect liver activity	
		Rosmarinci acid		
		Trans- cinnamic acid		
		Myricetin	Liver protection and lowering enzymes, anti-atherosclerosis, antibacterial and viral infection, anti-allergy	
		Linalool	Anti-inflammatory, antimicrobial, sedative effects, improve fat quality	
	Banana	Osides	Cholesterol lowering, anti-inflammatory, bacteriostatic, and immune regulation	[26]
		Cellulose	Promotes digestive health, prevent constipation	
		Ferulic acid	Antioxidant, anti-inflammatory, anticarcinogenic	
	Grape	Procatechin acid	Antiplatelet agglutination, improve myocardial oxygen resistance, bacteriostasis, analgesia, anti-inflammatory	[22]
		Catechin	Antioxidant, cardiovascular health, anticarcinogenic	
		Trans cassia bark acid	Anti-inflammatory, antimicrobial, antidiabetic, hemostasia	
		Resveratrol	Cardiovascular protection, anticarcinogenic, anti-aging	
		Condensed tannins	Antioxidant, anti-inflammatory, astringent	
	Amaranth	Vitamin C	Immune system support, antioxidant, promotes collagen synthesis	[20]
		Vitamin E	Antioxidant, protects cell membranes, supports skin and eye health, prevention of atherosclerosis	
		Vitamin B2	Promotes cell regeneration, supports energy metabolism	
		Carotenoids	Antioxidant, supports eye health, may reduce risk of certain cancers	
		Saponins	Anti-diabetic properties, lowers blood lipids	
	Broccoli	Glucosinolate	Anticancer, anti-inflammatory, regulation of the endocrine function, protect the liver, protect the renal function, analgesic	[27]
		Sulforaphen		
		Polyphenols	Antioxidant, Vision health, Immune support	
		Carotenoids		
		Chlorophyll		
		Vitamin E	Antioxidant, Supports immune function	
		Vitamin K	Blood clotting, Bone health	
Leaves	Celery	Vitamin C	Immune system support, antioxidant, promotes collagen synthesis, aiding in iron absorption	[28]
		Chlorophyll	Antioxidant, antimutagenic, antigenotoxic, anticancer, anti-obesogenic activities	
		Quercetin	Preventing allergies, preventing free radicals, supporting cardiovascular health, expansion of the coronary artery	
		Kaempferol	Antioxidant, anti-inflammatory, anticancer, prevention of arteriosclerosis, improve insulin sensitivity	
		Apigenin	Anti-tumor, cardiovascular and cerebrovascular protection, antiviral, antibacterial, support mental health	
		Luteolin	Dispellant, anti-inflammatory, uric acid, anti-allergy, regulation of aging	
	Grape	Galactool	Prebiotic effect, promotes beneficial gut bacteria	[29]
		Raffinose	Promotes the growth of beneficial bacteria, reduces pathogens	
		Vitamin C	Antioxidant, boosts immunity, aids in collagen synthesis	
		GABA	Reduces anxiety, improves sleep quality, lowers blood pressure, mediation of hormone secretion	
	Sea-buckthorn	Ellagitannins	Antioxidant, anti-inflammatory, chemopreventive, prevention of neurodegenerative diseases	[30]
		GABA	Reduces anxiety, improves sleep quality, lowers blood pressure, mediation of hormone secretion	
		carotenoids	Antioxidation, eye health, cardiovascular protection, anticancer	
		Flavonoids		
		Gallic acid	Antioxidant, anti-inflammatory, and antineoplastic properties	
		Ellagic acid	Boosts immune system, lowers cholesterol, helps prevent heart disease	
	Soybean	Ceramphenolic glycosides	Anti-infection, anti-inflammatory disease, prevention of diabetes mellitus	[31]
		Daidzin	Anti-estrogenic effects, breast cancer, and cardiovascular disease, antiatherosclerotic activities	
		Coumesterol	Reduce the oxidation of low-density lipoprotein, prevent cancer and anti-inflammatory effects	
		Glyceofuran	Antiestrogenic effects, antifungal activity against Aspergillus	
		Pterocarpan	Hyglucose glycemia, anti-inflammatory, antioxidant, analgesia	
Seeds	Tamarindus	Ligninans	Antioxidant, anticancer, hepatic protection and estrogenic properties, anantagonism of platelet activating factor activity	[32]
		Xyloglucan	Immunomodulatory activity	
		Dietary fiber	Helps regulate blood sugar, aids in weight management, promotes heart health	
		Flavonols	Antioxidant, anti-inflammatory, anticancer, and cardioprotective effects	
	Cactus	Hydroxyl cassia bark acid	Anti-inflammatory, antidiabetic, anticancer, antioxidant activity	[33]
		Linoleic acid	Anticarcinogenic, antiobese, antidiabetic and antihypertensive properties	
		Oleic acid	Promotes health and longevity, involved in lipid metabolism	
		Tocopherol	Antioxidant, prevents certain types of cancer, heart disease, and other chronic ailments	
	Pomegranate	Punicic acid	Cardiovascular health, anti-inflammatory, antioxidant	[34]
	Watermelon	Linoleic acid	Anticarcinogenic, antiobese, antidiabetic and antihypertensive properties	[32]
		Oleic acid	Promotes health and longevity, involved in lipid metabolism	
		Carotenoids	Antioxidation, eye health, cardiovascular protection, anticancer	
	Foeniculum vulgare	Trans-anethole	Flavoring agent	[35]
		2-pentanone	Antibacterial, anti-inflammatory, analgesic, antioxidant liver protection	
		Fenchone		
		Benzaldehyde-4-methoxy		

**Table 3:** Sustainable extraction technology of bioactive substances and application in food.

Bioactivator	Extraction Method and Parameters			Novel Food Ingredients		
Polysaccharide	Gold kiwifruit (*Actinidia chinensis*)	EAE (25 °C for 30 min with Celluclast 1.5 L, contained a mixture of cellulase, polygalacturonase, and arabinase with activities of 50.81, 40.82, and 42.42 nkatals/mL, respectively)	Pectin (4.5% *w*/*w* dry matter)	[85]	Yogurt thickener	[86]
	*Hylocereus polyrhizus* peels	UAE (frequency of 132 kHz and power output of 80 W, 250 rpm with acid solutions 12 *v*/*w* at pH 2, temperature 65 °C for 70 min)	Pectin (31.04%, and pectin DE is 56.10%)	[87]	Potential emerging prebiotic	[86]
	*Rosa canina* peel	UAE (70.0 °C for 66 min, water to raw material ratio was 13 mL/g and ultrasound power was 230 W)	Polysaccharides (5.16 ± 1.81%)	[88]	Fat substitutes in sugar-free carbonated drinks	[86]
	Mung bean peel	UAE (solid–liquid ratio of 1:40, at 77 °C, ultrasonic power of 216 W, for 47 min)	Polysaccharide (extraction rate was 2.55%)	[89]	Anti-lipid peroxidation	[89]
	Banana peels	MAE (96% ethanol, 60 °C, 102 min, the liquid-solid ratio of 40% *v*/*w*, pH of 2.7)	Pectin yield and DE (14.34% and 63.58%)	[90]	Fat replacer in spreads, ice cream, fruit preparations for yogurt	[86]
	Berry pomace, pumpkin pulp, and beet pulp	EAE (nitric acid-assisted extraction, 2 h, 62.9%; enzymatic-assisted extraction, 12 h, 75.0%; enzymatic-assisted extraction, 48 h, 89.8%; and nitric acid-assisted extraction, 4 h, 76.5%, respectively)	Galactose polysaccharide (85.9%, 82.2% and 89.8%, respectively)	[91]	Heat-reversible bakery glazing,	[86]
	*Satsuma mandarin* peel	HHP (using two different solvents, citric acid and hydrochloric acid, ranged from 15.34% to 18.99%; at 500 MPa for 10 min)	Citric acid as an extractant, yield of pectin (18.99%)	[92]	Emulsified meat	[86]
	Beet	HHP (250, 350, and 450 MPa at 40 °C for 5 min with/without acid)	Pectin extraction rate (12.23 ± 0.13%)	[93]	Edible film	[94]
	Lemon peel	OHAE (11 V/cm, a solid–liquid ratio of 1:60 g/mL, and a retention time of 120 min)	Pectin yield of 18.51%	[95]	Unflocculant in acidified dairy beverages	[94]
Protein	Beer residue, coffee residue, and collard stems	MAE (110 °C, 10 min and 0.5 M NaOH)	Proteins (yields were 93.7%, 60.3%, and 95.4%, respectively; and the highest extraction was 14.6 kg protein/100 kg BSG DM)	[96]	Nutrient enhancer in high-protein pasta	[97]
	Broccoli leaves	MAE (80 ± 2 °C using an intermittent microwave device set for 450 Watt)	Crude protein content (34.40 m/m%)	[98]	Lower the saturated fatty acids in fish filets	[99]
	Oilseed rape stems and leaves (*Brassica napus* L.)	PEF (20 kV/cm)	Protein extraction rate (80%)	[100]	Increase the protein content in the beverage	[101]
	Tips, bases, and sheaths of bamboo shoots	DES (1:6 ratio of choline chloride to acetylpropanoic acid)	Protein (39.16 ± 1.22 mg/g DW)	[102]	Plant protein mix of infant formula	[103]
Polyphenol	Orange peels	SPE (10 MPa and 110–150 °C)	DOP (31.70 ± 1.46 AG/g)	[82]	Colorant of bread and dough	[104]
	Pomegranate peel	SPE (CO_2_, 2.5 h extraction at 40–50 °C and 20–30 MPa using 20% of the co-solvent)	TPC (6326 ± 414 mg GAE/100 g)	[105]	Natural antioxidants for freshly cut apples	[106]
	Papaya leaves	UAE (temperature of 62.84 °C, 19.98 min ultrasonication time and 0.2 g/mL)	TPC (149.12 mg GAE/g)	[107]	Flavoring agents in the beverage	[108]
	Rye bran	UAE (29 min, 66 °C, and solid–solvent ratio of 1:45 g/mL)	TPC (245.74 mg GAE/100 g DW)	[109]	Antioxidants for protein and oil in chicken intestines	[110]
	Eggplant peels (*Birgah*, *Black Bell* and *Black Moon*)	UAE (methanol containing 1% TFA, ultrasonic water bath for 30 min)	5-O-caffeoylquinic acid (6663 mg CAE kg/DM)	[111]	Nutrition enhanced capsules	[112]
	Eggplant peels	MAE (power, extraction time, liquid–solid ratio, ethanol concentration, and pH of solvent were 269.82 W, 7.98 min, 5.01 mL/g, 73.49%, and 3.06%, respectively)	Extraction rate, TPC, TFC, TAC (3.27%, 1049.84 μg GAE/mL, 130.40 μg QE/mL, 6.99 mg/L, respectively)	[111]	Oat paste coloring agent and nutrition enhancer	[113]
	Broccoli leaves	MAE (80% methanol, 10 min, and 73.27 °C)	TPC (1940.35 ± 0.794 μg GAE/g DW)	[5]	Bread seasoning agent	[114]
	Broccoli stems, leaves, and florets	MAE (74.54% methanol, 15.9 min, and 74.45 °C for broccoli stems; 80% methanol, 10 min, and 73.27 °C for broccoli leaves; and 80% methanol, 18.9 min, and 75 °C for broccoli florets)	TPC of leaves, florets and stems (1940.35 ± 0.794, 657.062 ± 0.771 and 225.273 ± 0.897 μg GAE/g DW)	[115]	Biological anti-corrosion coating	[116]
	Potato pericarp	PEF (1 kV/cm and 5 kJ/kg)	TPC (1180 mg GAE/kg FW)	[117]	Bacteriostasis agents in smoked pork sausages	[118]
	Cocoa bean shell and coffee silver skin	PEF (ethanol 50% and E = 1.2 kV/cm, electric field)	Main methylxanthines (theobromine and caffeine) and phenolic compounds (epigallocatechin and 5-caffeoylquinic acid), increased by 20% and 21.3%, respectively)	[119]	Potential metabolic prebiotics	[120]
	Cherry pomace	HHP (400 MPa to 500 MPa)	TPC (rose from 39.5% to 61%, with the final result of 227.51 ± 1.28 mg GAE/100 g FW)	[121]	Nutrient enhancer in yogurt	[122]
	Grape skins	DES (choline chloride and fructose in a 1.9:1 ratio, with the addition of 30% water)	TPC (100 mg/g dw and exhibited reduced cytotoxicity)	[123]	Improving gluten strength and noodle texture	[124]
	Dried mint leaves	CAP (50 W power for 20 min of plasma treatment)	TPC (292.53 mg GAE/g dw)	[125]	Natural preservative for chicken breast meat	[126]
	Rice and corn bran	CAP (220 V, 240 V, and 260 V for a treatment time ranging from 5 to 30 min)	Concentration of rice bran (containing vanillin, ferulic acid, sinapic acid, and chlorogenic acid) and corn bran (comprising 4-hydroxybenzaldehyde, p-coumaric acid, sinapic acid, and ferulic acid), was enhanced	[127]	Fresh-cut fruit disinfectant	[128]
Carotenoids	Corn kernels	SPE (SCC powder 5 g, extraction pressure was 350 bars, at 60 °C. CO_2_ was fed into the extraction vessel at 15 g/min for 1 h, with 15% ethanol)	Lutein, Zeaxanthin, β-carotene (3.81 ± 0.02, 8.47 ± 0.09, 177.29 ± 4.35, mg/kg DW, respectively)	[84]	Astaxanthin dietary supplement capsules	[129]
	Peels of sweet potato, tomato, apricot, pumpkin, peppers, and peach	SPE (CO_2_ at a flow rate of 15 g/min, the temperature of 59 °C, the pressure of 350 bar, and 15.5% ethanol)	More than 90% *w*/*w*, with the highest extraction rate of β-carotene, 80–100% *w*/*w*	[130]	Dough coloring agent	[129]
	Tomato peels	SPE (60 °C, CO_2_ at a flow rate of 2 mL/min, 550 bar for 80 min)	Lycopene (0.86 mg/100 g) and β-carotene (1.5 mg/100 g)	[131]	Biodegradable packaging thin film	[132]
	Tomato peel	PEF (in a mixture of hexane–acetone–ethanol with a 50:25:25 ratio, 3–7 kV/cm and 0–300 μs)	Carotenoid extraction rate increased by 39% compared with the control	[133]	As additives to enhance the stability of oils	[134]
	Tomato peels	EAE (a second enzymatic treatment using a protease cocktail)	Lycopene concentration (10% *w*/*w*, DW), increased 20–30 times over untreated tomato peels	[135]	Natural antioxidants for oils	[134]
	Papaya peels	HHP (with soybean oil as solvent, 400 MPa, 40.5 °C, 5 min)	Carotenoids (38.0 μg/g DW)	[136]	Antioxidant of apple juice	[137]
	Papaya seeds	HHP (using a medium concentration of ethanol at 500 Mpa, for 5–15 min)	Antioxidant extraction efficiencies (6.49 ± 0.06%), and when the pressurization time increased from 5 min to 15 min, the extraction rate increased from 9.8% to 20.4%	[138]	Shower in shrimp sauce	[139]
Flavonoids	*Herba Epimedii*	DES (L-proline and glycerol, DES–water ratio of 7:3, *v*/*v*, mixed by vortex for 5 min, followed by ultrasonic extraction at room temperature for 45 min)	Five main bioactive flavonoids were within the range of 88.5–107.7%	[140]	Bitter taste inhibitors and flavor improvements of traditional Chinese medicine	[141]
	Orange peels	UAE (under optimal conditions of 40 °C, 150 W and a ratio of 4:1 ethanol–water)	Naringenin and hesperidin (70.3 mg, 205.2 mg/100 g FW)	[142]	Bacteriostatic agent of fermented sausages	[12]
	Citrus peels	SPE (20% ethanol at 20 MPa, 20 °C and supercritical CO_2_ at 30 MPa, 60 °C)	Naringin (35.26, 44.05 and 19.86 mg/g in orange, tangerine and lemon peel extracts, respectively)	[143]	Antioxidants and bacteriostatic agents in minced chicken meat and apple juice	[144]
	Citrus peels	MAE (70% aqueous ethanol solution, microwave power used in the process was 1 kW at 2.45 GHz at 140 °C for 8 min)	Hesperidin, and extraction efficiency (47.7 mg/g, 86.8%)	[145]	Nutrient enhancer in soy bread, reducing prostate cancer risk factor	[146]

## Data Availability

No new data were created or analyzed in this study. Data sharing is not applicable to this article.

## Abbreviations

FVW	Fruit and vegetable waste
FAO	Food and Agriculture Organization
SDGs	Sustainable Development Goals
CAE	Catechin equivalent
DW	Dry weight
FW	Fresh weight
SCFAs	Short-chain fatty acids
DF	Dietary fiber
SPS	Soybean polysaccharides
GalA	Galacturonic acid
DE	Degree of esterification
BPs	Bioactive peptides
GAE	Gallic acid equivalent
TPC	Total phenolic content
TFC	Total flavonoid content
QE/mL	Quercetin equivalent per milliliter
TAC	Total antioxidant capacity
DM	Dry matter
TFA	Trifluoroacetic acid
DEC	Diethyl carbonate
2-MeTHF	2-methyltetrahydrofuran
GMP	Good Manufacturing Practice
IL	Ionic liquids
NADES	Natural Deep Eutectic Solvents
DES	Deep Eutectic Solvents
SPE	Supercritical fluid extraction
UAE	Ultrasound-assisted extraction
MAE	Microwave-assisted extraction
EAE	Enzyme-assisted extraction
PEFAE	Pulsed electric field-assisted extraction
HHPAE	High hydrostatic pressure-assisted extraction
OHAE	Ohmic heating-assisted extraction
DESAE	Deep eutectic solvent-assisted extraction
CAPPAE	Cold atmospheric pressure plasma-assisted extraction

## References

[1] Almeida J.M., Lima V.A., Giloni-Lima P.C., Knob A. (2015). Passion Fruit Peel as Novel Substrate for Enhanced β-Glucosidases Production by Penicillium Verruculosum: Potential of the Crude Extract for Biomass Hydrolysis. Biomass Bioenergy.

[2] Sirisompong W., Jirapakkul W., Klinkesorn U. (2011). Response Surface Optimization and Characteristics of Rambutan (*Nephelium lappaceum* L.) Kernel Fat by Hexane Extraction. LWT-Food Sci. Technol..

[3] Chen Y., Huang B., Huang M., Cai B. (2011). On the Preparation and Characterization of Activated Carbon from Mangosteen Shell. J. Taiwan Inst. Chem. Eng..

[4] Porat R., Lichter A., Terry L.A., Harker R., Buzby J. (2018). Postharvest Losses of Fruit and Vegetables during Retail and in Consumers’ Homes: Quantifications, Causes, and Means of Prevention. Postharvest Biol. Technol..

[5] Rodríguez García S.L., Raghavan V. (2022). Green Extraction Techniques from Fruit and Vegetable Waste to Obtain Bioactive Compounds—A Review. Crit. Rev. Food Sci. Nutr..

[6] Georgiev R., Kalaydzhiev H., Slavov A., Ivanova P., Uzunova G., Chalova V.I. (2022). Residual Waste After Protein Isolation From Ethanol-Treated Rapeseed Meal Has Physico-Chemical Properties for Functional Food Systems Formulation. Waste Biomass Valor.

[7] Blasi A., Verardi A., Sangiorgio P., Iulianelli A., Cassano A., Conidi C., Petrotos K. (2022). 3—The Zero-Waste Economy: From Food Waste to Industry. Membrane Engineering in the Circular Economy.

[8] Sharma M., Hussain S., Shalima T., Aav R., Bhat R. (2022). Valorization of Seabuckthorn Pomace to Obtain Bioactive Carotenoids: An Innovative Approach of Using Green Extraction Techniques (Ultrasonic and Microwave-Assisted Extractions) Synergized with Green Solvents (Edible Oils). Ind. Crops Prod..

[9] Rana A., Samtiya M., Dhewa T., Mishra V., Aluko R.E. (2022). Health Benefits of Polyphenols: A Concise Review. J. Food Biochem..

[10] Liu Y., Wei R., Tan Z., Chen G., Xu T., Liu Z., Xiong H., Chen C., Zhuang Y. (2024). Association between Dietary Fiber Intake and Peripheral Artery Disease in Hypertensive Patients. J. Health Popul. Nutr..

[11] Eggersdorfer M., Wyss A. (2018). Carotenoids in Human Nutrition and Health. Arch. Biochem. Biophys..

[12] Dang Y., Hao L., Li X., Sun Y., Pan D., Wu Z., Shen J. (2021). Inhibitory Mechanism of Chinese Herbal Medicine Extracts on Escherichia Coli and Its Application to Fermented-Bag Sausage. LWT.

[13] Ashokkumar V., Flora G., Sevanan M., Sripriya R., Chen W.H., Park J.-H., Rajesh Banu J., Kumar G. (2023). Technological Advances in the Production of Carotenoids and Their Applications—A Critical Review. Bioresour. Technol..

[14] Banerjee J., Singh R., Vijayaraghavan R., MacFarlane D., Patti A.F., Arora A. (2017). Bioactives from Fruit Processing Wastes: Green Approaches to Valuable Chemicals. Food Chem..

[15] Zhang Q.-W., Lin L.-G., Ye W.-C. (2018). Techniques for Extraction and Isolation of Natural Products: A Comprehensive Review. Chin. Med..

[16] Wohlt D., Schwarz E., Schieber A., Bader-Mittermaier S. (2021). Effects of Extraction Conditions on Banana Peel Polyphenol Oxidase Activity and Insights into Inactivation Kinetics Using Thermal and Cold Plasma Treatment. Foods.

[17] Singh B., Singh J.P., Kaur A., Singh N. (2020). Phenolic Composition, Antioxidant Potential and Health Benefits of Citrus Peel. Food Res. Int..

[18] Sagar N.A., Pareek S., Gonzalez-Aguilar G.A. (2020). Quantification of Flavonoids, Total Phenols and Antioxidant Properties of Onion Skin: A Comparative Study of Fifteen Indian Cultivars. J. Food Sci. Technol..

[19] Agnihotri S., Dobhal P., Ashfaqullah S., Oli S., Tamta S. (2024). Phytochemical Screening of *Zanthoxylum armatum* Roots and Exploring Its Polyphenol Content and Antioxidant Activity. Nat. Prod. Res..

[20] Leal C., Costa C.M., Barros A.I.R.N.A., Gouvinhas I. (2021). Assessing the Relationship Between the Phenolic Content and Elemental Composition of Grape (*Vitis vinifera* L.) Stems. Waste Biomass Valor.

[21] Łata B., Trampczynska A., Paczesna J. (2009). Cultivar Variation in Apple Peel and Whole Fruit Phenolic Composition. Sci. Hortic..

[22] Aviram M., Volkova N., Coleman R., Dreher M., Reddy M.K., Ferreira D., Rosenblat M. (2008). Pomegranate Phenolics from the Peels, Arils, and Flowers Are Antiatherogenic: Studies in Vivo in Atherosclerotic Apolipoprotein E-Deficient (E0) Mice and in Vitro in Cultured Macrophages and Lipoproteins. J. Agric. Food Chem..

[23] Makori S.I., Mu T.-H., Sun H.-N. (2022). Profiling of Polyphenols, Flavonoids and Anthocyanins in Potato Peel and Flesh from Four Potato Varieties. Potato Res..

[24] Tariq A., Sahar A., Usman M., Sameen A., Azhar M., Tahir R., Younas R., Issa Khan M. (2023). Extraction of Dietary Fiber and Polyphenols from Mango Peel and Its Therapeutic Potential to Improve Gut Health. Food Biosci..

[25] Amanat M., Reza M.S., Shuvo M.S.R., Ahmed K.S., Hossain H., Tawhid M., Saifuzzaman M., Islam M.S., Mazumder T., Islam M.A. (2021). *Zingiber Roseum* Rosc. Rhizome: A Rich Source of Hepatoprotective Polyphenols. Biomed. Pharmacother..

[26] Kandasamy S., Baggu C., Javagal M.R., Lingamallu J.R., Yenamandra V., Aradhya S.M. (2014). Antioxidant Properties of Isolated Compounds from Banana Rhizome. J. Food Sci..

[27] Liu M., Zhang L., Ser S.L., Cumming J.R., Ku K.-M. (2018). Comparative Phytonutrient Analysis of Broccoli By-Products: The Potentials for Broccoli By-Product Utilization. Molecules.

[28] Ikewuchi J.C., Ikewuchi C.C., Ifeanacho M.O. (2019). Nutrient and Bioactive Compounds Composition of the Leaves and Stems of Pandiaka Heudelotii: A Wild Vegetable. Heliyon.

[29] Golubkina N.A., Kharchenko V.A., Moldovan A.I., Koshevarov A.A., Zamana S., Nadezhkin S., Soldatenko A., Sekara A., Tallarita A., Caruso G. (2020). Yield, Growth, Quality, Biochemical Characteristics and Elemental Composition of Plant Parts of Celery Leafy, Stalk and Root Types Grown in the Northern Hemisphere. Plants.

[30] Rossouw G.C., Orchard B.A., Šuklje K., Smith J.P., Barril C., Deloire A., Holzapfel B.P. (2017). Vitis Vinifera Root and Leaf Metabolic Composition during Fruit Maturation: Implications of Defoliation. Physiol. Plant..

[31] Ryu R., Jeong T.-S., Kim Y.J., Choi J.-Y., Cho S.-J., Kwon E.-Y., Jung U.J., Ji H.-S., Shin D.-H., Choi M.-S. (2016). Beneficial Effects of Pterocarpan-High Soybean Leaf Extract on Metabolic Syndrome in Overweight and Obese Korean Subjects: Randomized Controlled Trial. Nutrients.

[32] Al-Sayed H.M.A., Ahmed A.R. (2013). Utilization of Watermelon Rinds and Sharlyn Melon Peels as a Natural Source of Dietary Fiber and Antioxidants in Cake. Ann. Agric. Sci..

[33] Chbani M., El Harkaoui S., Willenberg I., Matthäus B. (2023). Review: Analytical Extraction Methods, Physicochemical Properties and Chemical Composition of Cactus (*Opuntia Ficus-Indica)* Seed Oil and Its Biological Activity. Food Rev. Int..

[34] Goula A.M., Adamopoulos K.G. (2012). A Method for Pomegranate Seed Application in Food Industries: Seed Oil Encapsulation. Food Bioprod. Process..

[35] Alam P., Abdel-Kader M.S., Alqarni M.H., Zaatout H.H., Ahamad S.R., Shakeel F. (2019). Chemical Composition of Fennel Seed Extract and Determination of Fenchone in Commercial Formulations by GC–MS Method. J. Food Sci. Technol..

[36] Mithul Aravind S., Wichienchot S., Tsao R., Ramakrishnan S., Chakkaravarthi S. (2021). Role of Dietary Polyphenols on Gut Microbiota, Their Metabolites and Health Benefits. Food Res. Int..

[37] Yamagata K. (2019). Polyphenols Regulate Endothelial Functions and Reduce the Risk of Cardiovascular Disease. Curr. Pharm. Des..

[38] Ding S., Jiang H., Fang J. (2018). Regulation of Immune Function by Polyphenols. J. Immunol. Res..

[39] Shimizu M. (2017). Multifunctions of Dietary Polyphenols in the Regulation of Intestinal Inflammation. J. Food Drug Anal..

[40] Kilua A., Nagata R., Han K.-H., Fukushima M. (2022). Beneficial Health Effects of Polyphenols Metabolized by Fermentation. Food Sci. Biotechnol..

[41] Luca S.V., Macovei I., Bujor A., Miron A., Skalicka-Woźniak K., Aprotosoaie A.C., Trifan A. (2020). Bioactivity of Dietary Polyphenols: The Role of Metabolites. Crit. Rev. Food Sci. Nutr..

[42] Dobrinčić A., Repajić M., Garofulić I.E., Tuđen L., Dragović-Uzelac V., Levaj B. (2020). Comparison of Different Extraction Methods for the Recovery of Olive Leaves Polyphenols. Processes.

[43] Soycan G., Schär M.Y., Kristek A., Boberska J., Alsharif S.N.S., Corona G., Shewry P.R., Spencer J.P.E. (2019). Composition and Content of Phenolic Acids and Avenanthramides in Commercial Oat Products: Are Oats an Important Polyphenol Source for Consumers?. Food Chem. X.

[44] Lebaka V.R., Wee Y.-J., Ye W., Korivi M. (2021). Nutritional Composition and Bioactive Compounds in Three Different Parts of Mango Fruit. Int. J. Environ. Res. Public Health.

[45] Kaeswurm J.A.H., Burandt M.R., Mayer P.S., Straub L.V., Buchweitz M. (2022). Bioaccessibility of Apple Polyphenols from Peel and Flesh during Oral Digestion. J. Agric. Food Chem..

[46] Alim A., Li T., Nisar T., Ren D., Zhai X., Pang Y., Yang X. (2019). Antioxidant, Antimicrobial, and Antiproliferative Activity-Based Comparative Study of Peel and Flesh Polyphenols from Actinidia Chinensis. Food Nutr. Res..

[47] Riaz A., Aadil R.M., Amoussa A.M.O., Bashari M., Abid M., Hashim M.M. (2021). Application of Chitosan-Based Apple Peel Polyphenols Edible Coating on the Preservation of Strawberry (*Fragaria ananassa* cv Hongyan) Fruit. J. Food Process. Preserv..

[48] Szymanowska U., Baraniak B. (2019). Antioxidant and Potentially Anti-Inflammatory Activity of Anthocyanin Fractions from Pomace Obtained from Enzymatically Treated Raspberries. Antioxidants.

[49] Tremocoldi M.A., Rosalen P.L., Franchin M., Massarioli A.P., Denny C., Daiuto É.R., Paschoal J.A.R., Melo P.S., de Alencar S.M. (2018). Exploration of Avocado By-Products as Natural Sources of Bioactive Compounds. PLoS ONE.

[50] Phumat P., Chaichit S., Potprommanee S., Preedalikit W., Sainakham M., Poomanee W., Chaiyana W., Kiattisin K. (2023). Influence of Benincasa Hispida Peel Extracts on Antioxidant and Anti-Aging Activities, Including Molecular Docking Simulation. Foods.

[51] Liu Q., Song B., Tong S., Yang Q., Zhao H., Guo J., Tian X., Chang R., Wu J. (2023). Research Progress on the Anticancer Activity of Plant Polysaccharides. Recent Patents Anti-Cancer Drug Discov..

[52] Govindarajan S., Ayesha N. (2021). Biological Activities of Plant Polysaccharides, Mechanism of Action and Biomedical Applications. Res. J. Biotechnol..

[53] Ji X., Peng Q., Wang M. (2018). Anti-Colon-Cancer Effects of Polysaccharides: A Mini-Review of the Mechanisms. Int. J. Biol. Macromol..

[54] Ali Q., Ma S., La S., Guo Z., Liu B., Gao Z., Farooq U., Wang Z., Zhu X., Cui Y. (2022). Microbial Short-Chain Fatty Acids: A Bridge between Dietary Fibers and Poultry Gut Health—A Review. Anim. Biosci..

[55] Wu D., Ye X., Linhardt R.J., Liu X., Zhu K., Yu C., Ding T., Liu D., He Q., Chen S. (2021). Dietary Pectic Substances Enhance Gut Health by Its Polycomponent: A Review. Comp. Rev. Food Sci. Food Saf..

[56] Felisberto M.H.F., Beraldo A.L., Sentone D.T., Klosterhoff R.R., Clerici M.T.P.S., Cordeiro L.M.C. (2021). Young Culm of *Dendrocalamus asper*, *Bambusa tuldoides* and *B. vulgaris* as Source of Hemicellulosic Dietary Fibers for the Food Industry. Food Res. Int..

[57] Chaudhari K.V., Chaudhari D.P., Hamid H.S. (2023). Extraction, Identification, Evaluation and Comparative Study of Pectin Obtained from Various Natural Sources. IJRASET.

[58] Minjares-Fuentes R., Femenia A., Comas-Serra F., Rodríguez-González V.M. (2018). Compositional and Structural Features of the Main Bioactive Polysaccharides Present in the *Aloe vera* Plant. J. AOAC Int..

[59] Jia X., Chen M., Wan J.-B., Su H., He C. (2015). Review on the Extraction, Characterization and Application of Soybean Polysaccharide. RSC Adv..

[60] İnan Ö., Özcan M.M., Aljuhaimi F. (2018). Effect of Location and *Citrus* Species on Total Phenolic, Antioxidant, and Radical Scavenging Activities of Some *Citrus* Seed and Oils. J. Food Process. Preserv..

[61] Grzelak-Błaszczyk K., Karlińska E., Grzęda K., Rój E., Kołodziejczyk K. (2017). Defatted Strawberry Seeds as a Source of Phenolics, Dietary Fiber and Minerals. LWT.

[62] Sagar N.A., Pareek S., Sharma S., Yahia E.M., Lobo M.G. (2018). Fruit and Vegetable Waste: Bioactive Compounds, Their Extraction, and Possible Utilization. Comp. Rev. Food Sci. Food Saf..

[63] Petkowicz C.L.O., Williams P.A. (2020). Pectins from Food Waste: Characterization and Functional Properties of a Pectin Extracted from Broccoli Stalk. Food Hydrocoll..

[64] Olennikov D.N., Chemposov V.V., Chirikova N.K. (2022). Polymeric Compounds of Lingonberry Waste: Characterization of Antioxidant and Hypolipidemic Polysaccharides and Polyphenol-Polysaccharide Conjugates from Vaccinium Vitis-Idaea Press Cake. Foods.

[65] Zhu Y., Li Y., Li X., Chen T., Zhao H., Zhou H. (2024). Activities of Polysaccharide Fractions from Corn Silk: Hemostatic, Immune, and Anti-Lung Cancer Potentials. Int. J. Biol. Macromol..

[66] Sibiya N.P., Kayitesi E., Moteetee A.N. (2021). Proximate Analyses and Amino Acid Composition of Selected Wild Indigenous Fruits of Southern Africa. Plants.

[67] Daliri E.B.-M., Oh D.H., Lee B.H. (2017). Bioactive Peptides. Foods.

[68] Cicero A.F.G., Fogacci F., Colletti A. (2017). Potential Role of Bioactive Peptides in Prevention and Treatment of Chronic Diseases: A Narrative Review. Br. J. Pharmacol..

[69] Zhang X., Zhang Z., Shen A., Zhang T., Jiang L., El-Seedi H., Zhang G., Sui X. (2024). Legumes as an Alternative Protein Source in Plant-Based Foods: Applications, Challenges, and Strategies. Curr. Res. Food Sci..

[70] Gu M., Chen H.-P., Zhao M.-M., Wang X., Yang B., Ren J.-Y., Su G.-W. (2015). Identification of Antioxidant Peptides Released from Defatted Walnut (Juglans Sigillata Dode) Meal Proteins with Pancreatin. LWT-Food Sci. Technol..

[71] Prade T., Muneer F., Berndtsson E., Nynäs A.-L., Svensson S.-E., Newson W.R., Johansson E. (2021). Protein Fractionation of Broccoli (*Brassica oleracea*, Var. Italica) and Kale (*Brassica oleracea*, Var. Sabellica) Residual Leaves—A Pre-Feasibility Assessment and Evaluation of Fraction Phenol and Fibre Content. Food Bioprod. Process..

[72] Kumoro A.C., Alhanif M., Wardhani D.H. (2020). A Critical Review on Tropical Fruits Seeds as Prospective Sources of Nutritional and Bioactive Compounds for Functional Foods Development: A Case of Indonesian Exotic Fruits. Int. J. Food Sci..

[73] Islam F., Khan J., Zehravi M., Das R., Haque M.A., Banu A., Parwaiz S., Nainu F., Nafady M.H., Shahriar S.M.S. (2024). Synergistic Effects of Carotenoids: Therapeutic Benefits on Human Health. Process Biochem..

[74] Hanschen E.R., Starkenburg S.R. (2020). The State of Algal Genome Quality and Diversity. Algal Res..

[75] Cassani L., Marcovich N.E., Gomez-Zavaglia A. (2022). Valorization of Fruit and Vegetables Agro-Wastes for the Sustainable Production of Carotenoid-Based Colorants with Enhanced Bioavailability. Food Res. Int..

[76] Silva Y.P.A., Ferreira T.A.P.C., Jiao G., Brooks M.S. (2019). Sustainable Approach for Lycopene Extraction from Tomato Processing By-Product Using Hydrophobic Eutectic Solvents. J. Food Sci. Technol..

[77] Surano B., Leiva G., Marshall G., Maglietti F., Schebor C. (2022). Pulsed Electric Fields Using a Multiple Needle Chamber to Improve Bioactive Compounds Extraction from Unprocessed Opuntia Ficus-Indica Fruits. J. Food Eng..

[78] Urbanavičiūtė I., Rubinskiene M., Viškelis P. (2019). The Fatty Acid Composition and Quality of Oils from Post-Industrial Waste of Quince *Chaenomeles japonica*. Chem. Biodivers..

[79] Jahurul M.H.A., Zaidul I.S.M., Nik Norulaini N.A., Sahena F., Kamaruzzaman B.Y., Ghafoor K., Omar A.K.M. (2014). Cocoa Butter Replacers from Blends of Mango Seed Fat Extracted by Supercritical Carbon Dioxide and Palm Stearin. Food Res. Int..

[80] Ko M.-J., Cheigh C.-I., Chung M.-S. (2014). Relationship Analysis between Flavonoids Structure and Subcritical Water Extraction (SWE). Food Chem..

[81] Kupnik K., Leitgeb M., Primožič M., Postružnik V., Kotnik P., Kučuk N., Knez Ž., Marevci M.K. (2022). Supercritical Fluid and Conventional Extractions of High Value-Added Compounds from Pomegranate Peels Waste: Production, Quantification and Antimicrobial Activity of Bioactive Constituents. Plants.

[82] Lachos-Perez D., Baseggio A.M., Mayanga-Torres P.C., Maróstica M.R., Rostagno M.A., Martínez J., Forster-Carneiro T. (2018). Subcritical Water Extraction of Flavanones from Defatted Orange Peel. J. Supercrit. Fluids.

[83] Madhumeena S., Preetha R., Prasad S. (2021). Effective Utilization of Pineapple Waste. J. Phys. Conf. Ser..

[84] Lau T., Harbourne N., Oruña-Concha M.J. (2019). Valorisation of Sweet Corn (*Zea mays*) Cob by Extraction of Valuable Compounds. Int. J. Food Sci. Technol..

[85] Yuliarti O., Goh K.K.T., Matia-Merino L., Mawson J., Brennan C. (2015). Extraction and Characterisation of Pomace Pectin from Gold Kiwifruit (*Actinidia chinensis*). Food Chem..

[86] Marić M., Grassino A.N., Zhu Z., Barba F.J., Brnčić M., Rimac Brnčić S. (2018). An Overview of the Traditional and Innovative Approaches for Pectin Extraction from Plant Food Wastes and By-Products: Ultrasound-, Microwaves-, and Enzyme-Assisted Extraction. Trends Food Sci. Technol..

[87] Zaid R.M., Mishra P., Siti Noredyani A.R., Tabassum S., Ab Wahid Z., Mimi Sakinah A.M. (2020). Proximate Characteristics and Statistical Optimization of Ultrasound-Assisted Extraction of High-Methoxyl-Pectin from *Hylocereus Polyrhizus* Peels. Food Bioprod. Process..

[88] Wang S., Li S., Zhao H., Gu P., Chen Y., Zhang B., Zhu B. (2018). Acetaldehyde Released by Lactobacillus Plantarum Enhances Accumulation of Pyranoanthocyanins in Wine during Malolactic Fermentation. Food Res. Int..

[89] Zhang W., Duan W., Huang G., Huang H. (2023). Ultrasonic-Assisted Extraction, Analysis and Properties of Mung Bean Peel Polysaccharide. Ultrason. Sonochem..

[90] Aklilu E.G. (2021). Modeling and Optimization of Pectin Extraction from Banana Peel Using Artificial Neural Networks (ANNs) and Response Surface Methodology (RSM). Food Meas..

[91] Babbar N., Roy S.V., Wijnants M., Dejonghe W., Caligiani A., Sforza S., Elst K. (2016). Effect of Extraction Conditions on the Saccharide (Neutral and Acidic) Composition of the Crude Pectic Extract from Various Agro-Industrial Residues. J. Agric. Food Chem..

[92] Duan X., Zhu Y., Shu C., Gao J., Liu F., Pan S. (2022). Extraction of Pectin from Satsuma Mandarin Peel: A Comparison of High Hydrostatic Pressure and Conventional Extractions in Different Acids. Molecules.

[93] Kaya B., Okur I., Alpas H., Oztop M.H. (2021). High Hydrostatic Pressure Assisted Extraction of Pectin from Sugar Beet Pulp. Int. J. Food Sci. Technol..

[94] Belkheiri A., Forouhar A., Ursu A.V., Dubessay P., Pierre G., Delattre C., Djelveh G., Abdelkafi S., Hamdami N., Michaud P. (2021). Extraction, Characterization, and Applications of Pectins from Plant By-Products. Appl. Sci..

[95] Çilingir S., Duran G., Gökyildiz B., Goksu A., Sabanci S., Cevik M. (2024). Optimization of Pectin Extraction from Lemon Peel Powder by Ohmic Heating Using Full Factorial Design. Food Bioprocess Technol..

[96] Barrios C., Fernández-Delgado M., López-Linares J.C., García-Cubero M.T., Coca M., Lucas S. (2022). A Techno-Economic Perspective on a Microwave Extraction Process for Efficient Protein Recovery from Agri-Food Wastes. Ind. Crops Prod..

[97] Filip S., Vidrih R. (2015). Amino Acid Composition of Protein-Enriched Dried Pasta: Is It Suitable for a Low-Carb Diet?. Food Technol. Biotechnol..

[98] Domokos-Szabolcsy É., Elhawat N., Domingos G.J., Kovács Z., Koroknai J., Bodó E., Fári M.G., Alshaal T., Bákonyi N. (2022). Comparison of Wet Fractionation Methods for Processing Broccoli Agricultural Wastes and Evaluation of the Nutri-Chemical Values of Obtained Products. Foods.

[99] Bruni L., Secci G., Mancini S., Faccenda F., Parisi G. (2020). A Commercial Macroalgae Extract in a Plant-Protein Rich Diet Diminished Saturated Fatty Acids of *Oncorhynchus mykiss* Walbaum Fillets. Ital. J. Anim. Sci..

[100] Yu X., Bals O., Grimi N., Vorobiev E. (2015). A New Way for the Oil Plant Biomass Valorization: Polyphenols and Proteins Extraction from Rapeseed Stems and Leaves Assisted by Pulsed Electric Fields. Ind. Crops Prod..

[101] Abbaschian S., Soltani M. (2024). Functional, Structural, and Rheological Properties of the Complexes Containing Sunflower Petal Extract with Dairy and Plant-Based Proteins. Food Chem..

[102] Lin Z., Jiao G., Zhang J., Celli G.B., Brooks M.S.-L. (2021). Optimization of Protein Extraction from Bamboo Shoots and Processing Wastes Using Deep Eutectic Solvents in a Biorefinery Approach. Biomass Convers. Bioref..

[103] Roux L.L., Chacon R., Dupont D., Jeantet R., Deglaire A., Nau F. (2020). In Vitro Static Digestion Reveals How Plant Proteins Modulate Model Infant Formula Digestibility. Food Res. Int..

[104] Sowbhagya H.B., Chitra V.N. (2010). Enzyme-Assisted Extraction of Flavorings and Colorants from Plant Materials. Crit. Rev. Food Sci. Nutr..

[105] Rivas M.Á., Casquete R., de Guía Córdoba M., Benito M.J., Hernández A., Ruiz-Moyano S., Martín A. (2021). Functional Properties of Extracts and Residual Dietary Fibre from Pomegranate (*Punica granatum* L.) Peel Obtained with Different Supercritical Fluid Conditions. LWT.

[106] Martínez-Hernández G.B., Castillejo N., Artés-Hernández F. (2019). Effect of Fresh–Cut Apples Fortification with Lycopene Microspheres, Revalorized from Tomato by-Products, during Shelf Life. Postharvest Biol. Technol..

[107] Wani K.M., Uppaluri R.V.S. (2023). Comparative Efficacy of Ultrasound-Assisted and Hot Water Extraction of Papaya Leaves. J. Herb. Med..

[108] Meena L., Sengar A.S., Neog R., Sunil C.K. (2022). Pineapple Processing Waste (PPW): Bioactive Compounds, Their Extraction, and Utilisation: A Review. J. Food Sci. Technol..

[109] Iftikhar M., Zhang H., Iftikhar A., Raza A., Begum N., Tahamina A., Syed H., Khan M., Wang J. (2020). Study on Optimization of Ultrasonic Assisted Extraction of Phenolic Compounds from Rye Bran. LWT.

[110] Manzoor A., Ahmad S., Yousuf B. (2022). Effect of Bioactive-Rich Mango Peel Extract on Physicochemical, Antioxidant and Functional Characteristics of Chicken Sausage. Appl. Food Res..

[111] Mauro R.P., Agnello M., Rizzo V., Graziani G., Fogliano V., Leonardi C., Giuffrida F. (2020). Recovery of Eggplant Field Waste as a Source of Phytochemicals. Sci. Hortic..

[112] Castaldo L., Izzo L., Gaspari A., Lombardi S., Rodríguez-Carrasco Y., Narváez A., Grosso M., Ritieni A. (2021). Chemical Composition of Green Pea (*Pisum sativum* L.) Pods Extracts and Their Potential Exploitation as Ingredients in Nutraceutical Formulations. Antioxidants.

[113] Pinho L.S., Patel B.K., Campanella O.H., Rodrigues C.E.D.C., Favaro-Trindade C.S. (2023). Microencapsulation of Carotenoid-Rich Extract from Guaraná Peels and Study of Microparticle Functionality through Incorporation into an Oatmeal Paste. Foods.

[114] Kessler J.C., Vieira V., Martins I.M., Manrique Y.A., Ferreira P., Calhelha R.C., Afonso A., Barros L., Rodrigues A.E., Dias M.M. (2023). The Potential of Almonds, Hazelnuts, and Walnuts SFE-CO_2_ Extracts as Sources of Bread Flavouring Ingredients. Food Chem..

[115] Rodríguez García S.L., Raghavan V. (2022). Microwave-Assisted Extraction of Phenolic Compounds from Broccoli (*Brassica oleracea*) Stems, Leaves, and Florets: Optimization, Characterization, and Comparison with Maceration Extraction. Recent Prog. Nutr..

[116] Li X., Gao P., Tan J., Xiong K., Maitz M.F., Pan C., Wu H., Chen Y., Yang Z., Huang N. (2018). Assembly of Metal–Phenolic/Catecholamine Networks for Synergistically Anti-Inflammatory, Antimicrobial, and Anticoagulant Coatings. ACS Appl. Mater. Interfaces.

[117] Frontuto D., Carullo D., Harrison S.M., Brunton N.P., Ferrari G., Lyng J.G., Pataro G. (2019). Optimization of Pulsed Electric Fields-Assisted Extraction of Polyphenols from Potato Peels Using Response Surface Methodology. Food Bioprocess Technol..

[118] Nicorescu V., Papuc C., Predescu C., Gajaila I., Petcu C., Stefan G. (2018). The Influence of Rosehip Polyphenols on the Quality of Smoked Pork Sausages, Compared to Classic Additives. Rev. Chim..

[119] Barbosa-Pereira L., Guglielmetti A., Zeppa G. (2018). Pulsed Electric Field Assisted Extraction of Bioactive Compounds from Cocoa Bean Shell and Coffee Silverskin. Food Bioprocess Technol..

[120] Nazzaro F., Fratianni F., De Feo V., Battistelli A., Da Cruz A.G., Coppola R. (2020). Polyphenols, the New Frontiers of Prebiotics. Adv. Food Nutr. Res..

[121] Okur İ., Baltacıoğlu C., Ağçam E., Baltacıoğlu H., Alpas H. (2019). Evaluation of the Effect of Different Extraction Techniques on Sour Cherry Pomace Phenolic Content and Antioxidant Activity and Determination of Phenolic Compounds by FTIR and HPLC. Waste Biomass Valoriz..

[122] Pimpley V.A., Maity S., Murthy P.S. (2022). Green Coffee Polyphenols in Formulations of Functional Yoghurt and Their Quality Attributes. Int. J. Dairy Technol..

[123] Radošević K., Ćurko N., Gaurina Srček V., Cvjetko Bubalo M., Tomašević M., Kovačević Ganić K., Radojčić Redovniković I. (2016). Natural Deep Eutectic Solvents as Beneficial Extractants for Enhancement of Plant Extracts Bioactivity. LWT.

[124] Han C.-W., Ma M., Zhang H.-H., Li M., Sun Q.-J. (2020). Progressive Study of the Effect of Superfine Green Tea, Soluble Tea, and Tea Polyphenols on the Physico-Chemical and Structural Properties of Wheat Gluten in Noodle System. Food Chem..

[125] Kashfi A.S., Ramezan Y., Khani M.R. (2020). Simultaneous Study of the Antioxidant Activity, Microbial Decontamination and Color of Dried Peppermint (*Mentha piperita* L.) Using Low Pressure Cold Plasma. LWT.

[126] Al-Hijazeen M., Lee E.J., Mendonca A., Ahn D.U. (2016). Effects of Tannic Acid on Lipid and Protein Oxidation, Color, and Volatiles of Raw and Cooked Chicken Breast Meat during Storage. Antioxidants.

[127] Mehta D., Yadav K., Chaturvedi K., Shivhare U.S., Yadav S.K. (2022). Impact of Cold Plasma on Extraction of Polyphenol From De-Oiled Rice and Corn Bran: Improvement in Extraction Efficiency, In Vitro Digestibility, Antioxidant Activity, Cytotoxicity and Anti-Inflammatory Responses. Food Bioprocess Technol..

[128] Martinez-Hernandez G.B., Amodio M.L., Colelli G. (2017). Carvacrol-Loaded Chitosan Nanoparticles Maintain Quality of Fresh-Cut Carrots. Innov. Food Sci. Emerg. Technol..

[129] López G.-D., Álvarez-Rivera G., Carazzone C., Ibáñez E., Leidy C., Cifuentes A. (2023). Bacterial Carotenoids: Extraction, Characterization, and Applications. Crit. Rev. Anal. Chem..

[130] de Andrade Lima M., Kestekoglou I., Charalampopoulos D., Chatzifragkou A. (2019). Supercritical Fluid Extraction of Carotenoids from Vegetable Waste Matrices. Molecules.

[131] Pellicanò T.M., Sicari V., Loizzo M.R., Leporini M., Falco T., Poiana M. (2020). Optimizing the Supercritical Fluid Extraction Process of Bioactive Compounds from Processed Tomato Skin By-Products. Food Sci. Technol..

[132] Medina-Jaramillo C., Ochoa-Yepes O., Bernal C., Famá L. (2017). Active and Smart Biodegradable Packaging Based on Starch and Natural Extracts. Carbohydr. Polym..

[133] Luengo E., Álvarez I., Raso J. (2014). Improving Carotenoid Extraction from Tomato Waste by Pulsed Electric Fields. Front. Nutr..

[134] Mercadante A.Z., Rodrigues D.B., Petry F.C., Mariutti L.R.B. (2017). Carotenoid Esters in Foods—A Review and Practical Directions on Analysis and Occurrence. Food Res. Int..

[135] Cuccolini S., Aldini A., Visai L., Daglia M., Ferrari D. (2013). Environmentally Friendly Lycopene Purification from Tomato Peel Waste: Enzymatic Assisted Aqueous Extraction. J. Agric. Food Chem..

[136] Lara-Abia S., Gomez-Maqueo A., Welti-Chanes J., Cano M.P. (2021). High Hydrostatic Pressure-Assisted Extraction of Carotenoids from Papaya (*Carica papaya* L. Cv. Maradol) Tissues Using Soybean and Sunflower Oil as Potential Green Solvents. Food Eng. Rev..

[137] Li J., Li Y., Zhang X., Miao S., Tan M., Su W. (2022). Microfluidic Spinning of Fucoxanthin-Loaded Nanofibers for Enhancing Antioxidation and Clarification of Fruit Juice. Food Funct..

[138] Briones-Labarca V., Plaza-Morales M., Giovagnoli-Vicuña C., Jamett F. (2015). High Hydrostatic Pressure and Ultrasound Extractions of Antioxidant Compounds, Sulforaphane and Fatty Acids from Chilean Papaya (*Vasconcellea pubescens*) Seeds: Effects of Extraction Conditions and Methods. LWT-Food Sci. Technol..

[139] Zahrah Z., Amin M.N.G., Alamsjah M.A. (2020). The Effect of Fucoxanthin as Coloring Agent on the Quality of Shrimp Paste. IOP Conf. Ser. Earth Environ. Sci..

[140] Guo H., Liu S., Li S., Feng Q., Ma C., Zhao J., Xiong Z. (2020). Deep Eutectic Solvent Combined with Ultrasound-Assisted Extraction as High Efficient Extractive Media for Extraction and Quality Evaluation of Herba Epimedii. J. Pharm. Biomed. Anal..

[141] Huang J., Lu Y., Guo C., Zuo S., Zhou J., Wong W., Huang B. (2021). The Study of Citrus-derived Flavonoids as Effective Bitter Taste Inhibitors. J. Sci. Food Agric..

[142] Khan M.K., Abert-Vian M., Fabiano-Tixier A.-S., Dangles O., Chemat F. (2010). Ultrasound-Assisted Extraction of Polyphenols (Flavanone Glycosides) from Orange (*Citrus sinensis* L.) Peel. Food Chem..

[143] Romano R., De Luca L., Aiello A., Rossi D., Pizzolongo F., Masi P. (2022). Bioactive Compounds Extracted by Liquid and Supercritical Carbon Dioxide from Citrus Peels. Int. J. Food Sci. Technol..

[144] Bajpai V.K., Park I., Lee J., Shukla S., Nile S.H., Chun H.S., Khan I., Oh S.Y., Lee H., Huh Y.S. (2019). Antioxidant and Antimicrobial Efficacy of a Biflavonoid, Amentoflavone from *Nandina domestica* in Vitro and in Minced Chicken Meat and Apple Juice Food Models. Food Chem..

[145] Inoue T., Tsubaki S., Ogawa K., Onishi K., Azuma J. (2010). Isolation of Hesperidin from Peels of Thinned Citrus Unshiu Fruits by Microwave-Assisted Extraction. Food Chem..

[146] Ahn-Jarvis J.H., Clinton S.K., Grainger E.M., Riedl K.M., Schwartz S.J., Lee M.-L.T., Cruz-Cano R., Young G.S., Lesinski G.B., Vodovotz Y. (2015). Isoflavone Pharmacokinetics and Metabolism after Consumption of a Standardized Soy and Soy–Almond Bread in Men with Asymptomatic Prostate Cancer. Cancer Prev. Res..

[147] Panzella L., Moccia F., Nasti R., Marzorati S., Verotta L., Napolitano A. (2020). Bioactive Phenolic Compounds From Agri-Food Wastes: An Update on Green and Sustainable Extraction Methodologies. Front. Nutr..

[148] Shen L., Pang S., Zhong M., Sun Y., Qayum A., Liu Y., Rashid A., Xu B., Liang Q., Ma H. (2023). A Comprehensive Review of Ultrasonic Assisted Extraction (UAE) for Bioactive Components: Principles, Advantages, Equipment, and Combined Technologies. Ultrason. Sonochem..

[149] Arvanitoyannis I.S., Kotsanopoulos K.V., Savva A.G. (2017). Use of Ultrasounds in the Food Industry–Methods and Effects on Quality, Safety, and Organoleptic Characteristics of Foods: A Review. Crit. Rev. Food Sci. Nutr..

[150] Tiwari B.K. (2015). Ultrasound: A Clean, Green Extraction Technology. TrAC Trends Anal. Chem..

[151] Corbin C., Fidel T., Leclerc E.A., Barakzoy E., Sagot N., Falguiéres A., Renouard S., Blondeau J.-P., Ferroud C., Doussot J. (2015). Development and Validation of an Efficient Ultrasound Assisted Extraction of Phenolic Compounds from Flax (*Linum usitatissimum* L.) Seeds. Ultrason. Sonochem..

[152] Wang Y., Wang F., Ma X., Sun S., Leng F., Zhang W., Wang X. (2015). Extraction, Purification, Characterization and Antioxidant Activity of Polysaccharides from Piteguo Fruit. Ind. Crops Prod..

[153] Ponmurugan K., Al-Dhabi N.A., Maran J.P., Karthikeyan K., Moothy I.G., Sivarajasekar N., Manoj J.J.B. (2017). Ultrasound Assisted Pectic Polysaccharide Extraction and Its Characterization from Waste Heads of Helianthus Annus. Carbohydr. Polym..

[154] Guandalini B.B.V., Rodrigues N.P., Marczak L.D.F. (2019). Sequential Extraction of Phenolics and Pectin from Mango Peel Assisted by Ultrasound. Food Res. Int..

[155] Freitas de Oliveira C., Giordani D., Lutckemier R., Gurak P.D., Cladera-Olivera F., Ferreira Marczak L.D. (2016). Extraction of Pectin from Passion Fruit Peel Assisted by Ultrasound. LWT-Food Sci. Technol..

[156] Ferrara D., Beccaria M., Cordero C.E., Purcaro G. (2023). Microwave-Assisted Extraction in Closed Vessel in Food Analysis. J. Sep. Sci..

[157] Chan C.-H., Yeoh H.K., Yusoff R., Ngoh G.C. (2016). A First-Principles Model for Plant Cell Rupture in Microwave-Assisted Extraction of Bioactive Compounds. J. Food Eng..

[158] Oualcadi Y., Sebban M.F., Berrekhis F. (2020). Improvement of Microwave-assisted Soxhlet Extraction of Bioactive Compounds Applied to Pomegranate Peels. J. Food Process. Preserv..

[159] Da Rosa G.S., Vanga S.K., Gariepy Y., Raghavan V. (2019). Comparison of Microwave, Ultrasonic and Conventional Techniques for Extraction of Bioactive Compounds from Olive Leaves (*Olea europaea* L.). Innov. Food Sci. Emerg. Technol..

[160] Doulabi M., Golmakani M.-T., Ansari S. (2020). Evaluation and Optimization of Microwave-Assisted Extraction of Bioactive Compounds from Eggplant Peel by-Product. J. Food Process. Preserv..

[161] Ho K.K.H.Y., Ferruzzi M.G., Liceaga A.M., San Martín-González M.F. (2015). Microwave-Assisted Extraction of Lycopene in Tomato Peels: Effect of Extraction Conditions on All-Trans and Cis-Isomer Yields. LWT-Food Sci. Technol..

[162] Terigar B.G., Balasubramanian S., Sabliov C.M., Lima M., Boldor D. (2011). Soybean and Rice Bran Oil Extraction in a Continuous Microwave System: From Laboratory- to Pilot-Scale. J. Food Eng..

[163] Chen Y., Zhang M., Mujumdar A.S., Liu Y. (2024). Study on the Regulation of Browning of Concentrated Orange Juice by Ultrasonic-Vacuum Combined with High-Pressure CO_2_ During Storage. Food Bioprocess Technol..

[164] Sheldon R.A., Van Pelt S. (2013). Enzyme Immobilisation in Biocatalysis: Why, What and How. Chem. Soc. Rev..

[165] Nadar S.S., Rao P., Rathod V.K. (2018). Enzyme Assisted Extraction of Biomolecules as an Approach to Novel Extraction Technology: A Review. Food Res. Int..

[166] Görgüç A., Gençdağ E., Yılmaz F.M. (2020). Bioactive Peptides Derived from Plant Origin By-Products: Biological Activities and Techno-Functional Utilizations in Food Developments—A Review. Food Res. Int..

[167] Montoya-Rodríguez A., Gómez-Favela M.A., Reyes-Moreno C., Milán-Carrillo J., González De Mejía E. (2015). Identification of Bioactive Peptide Sequences from Amaranth (*Amaranthus hypochondriacus*) Seed Proteins and Their Potential Role in the Prevention of Chronic Diseases. Comp. Rev. Food Sci. Food Saf..

[168] García M.C., González-García E., Vásquez-Villanueva R., Marina M.L. (2016). Apricot and Other Seed Stones: Amygdalin Content and the Potential to Obtain Antioxidant, Angiotensin I Converting Enzyme Inhibitor and Hypocholesterolemic Peptides. Food Funct..

[169] Ayala-Niño A., Rodríguez-Serrano G.M., González-Olivares L.G., Contreras-López E., Regal-López P., Cepeda-Saez A. (2019). Sequence Identification of Bioactive Peptides from Amaranth Seed Proteins (*Amaranthus hypochondriacus* spp.). Molecules.

[170] Ma N.B., Ton N.M.N., Le N.L. (2024). Co-Optimization of Polysaccharides and Polyphenols Extraction from Mangosteen Peels Using Ultrasound-Microwave Assisted Extraction (UMAE) and Enzyme-Ultrasound Assisted Extraction (EUAE) and Their Characterization. Food Meas..

[171] Su X., Xue Q., Sun M., Liu J., Wong M.H., Wang C., Chen S. (2021). Co-Production of Polysaccharides, Ginsenosides and Succinic Acid from Panax Ginseng Residue: A Typical Industrial Herbal Waste. Bioresour. Technol..

[172] Pataro G., Carullo D., Bakar Siddique M.A., Falcone M., Donsì F., Ferrari G. (2018). Improved Extractability of Carotenoids from Tomato Peels as Side Benefits of PEF Treatment of Tomato Fruit for More Energy-Efficient Steam-Assisted Peeling. J. Food Eng..

[173] Maza M.A., Martínez J.M., Delso C., Camargo A., Raso J., Álvarez I. (2020). PEF-Dependency on Polyphenol Extraction during Maceration/Fermentation of Grenache Grapes. Innov. Food Sci. Emerg. Technol..

[174] Le-Tan H., Fauster T., Vladic J., Gerhardt T., Haas K., Jaeger H. (2021). Application of Emerging Cell Disintegration Techniques for the Accelerated Recovery of Curcuminoids from *Curcuma longa*. Appl. Sci..

[175] Luengo E., Martínez J.M., Álvarez I., Raso J. (2016). Effects of Millisecond and Microsecond Pulsed Electric Fields on Red Beet Cell Disintegration and Extraction of Betanines. Ind. Crops Prod..

[176] Soquetta M.B., Terra L.D.M., Bastos C.P. (2018). Green Technologies for the Extraction of Bioactive Compounds in Fruits and Vegetables. CyTA-J. Food.

[177] Gharib-Bibalan S. (2018). High Value-Added Products Recovery from Sugar Processing By-Products and Residuals by Green Technologies: Opportunities, Challenges, and Prospects. Food Eng. Rev..

[178] Chiozzi V., Agriopoulou S., Varzakas T. (2022). Advances, Applications, and Comparison of Thermal (Pasteurization, Sterilization, and Aseptic Packaging) against Non-Thermal (Ultrasounds, UV Radiation, Ozonation, High Hydrostatic Pressure) Technologies in Food Processing. Appl. Sci..

[179] Moussa-Ayoub T.E., Jäger H., Knorr D., El-Samahy S.K., Kroh L.W., Rohn S. (2017). Impact of Pulsed Electric Fields, High Hydrostatic Pressure, and Thermal Pasteurization on Selected Characteristics of *Opuntia dillenii* Cactus Juice. LWT-Food Sci. Technol..

[180] Gómez-Maqueo A., Welti-Chanes J., Cano M.P. (2020). Release Mechanisms of Bioactive Compounds in Fruits Submitted to High Hydrostatic Pressure: A Dynamic Microstructural Analysis Based on Prickly Pear Cells. Food Res. Int..

[181] Morata A., Loira I., Vejarano R., Bañuelos M.A., Sanz P.D., Otero L., Suárez-Lepe J.A. (2015). Grape Processing by High Hydrostatic Pressure: Effect on Microbial Populations, Phenol Extraction and Wine Quality. Food Bioprocess Technol..

[182] Shouqin Z., Junjie Z., Changzhen W. (2004). Novel High Pressure Extraction Technology. Int. J. Pharm..

[183] Pereira R.N., Coelho M.I., Genisheva Z., Fernandes J.M., Vicente A.A., Pintado M.E., Teixeira J.A. (2020). Using Ohmic Heating Effect on Grape Skins as a Pretreatment for Anthocyanins Extraction. Food Bioprod. Process..

[184] Sabanci S., Çevik M., Göksu A. (2021). Investigation of Time Effect on Pectin Production from Citrus Wastes with Ohmic Heating Assisted Extraction Process. J. Food Process Eng..

[185] Saberian H., Hamidi-Esfahani Z., Ahmadi Gavlighi H., Barzegar M. (2017). Optimization of Pectin Extraction from Orange Juice Waste Assisted by Ohmic Heating. Chem. Eng. Process. Process Intensif..

[186] El Darra N., Grimi N., Vorobiev E., Louka N., Maroun R. (2013). Extraction of Polyphenols from Red Grape Pomace Assisted by Pulsed Ohmic Heating. Food Bioprocess Technol..

[187] Coelho M., Pereira R., Rodrigues A.S., Teixeira J.A., Pintado M.E. (2019). Extraction of Tomato By-Products’ Bioactive Compounds Using Ohmic Technology. Food Bioprod. Process..

[188] Al-Hilphy A.R., Al-Musafer A.M., Gavahian M. (2020). Pilot-Scale Ohmic Heating-Assisted Extraction of Wheat Bran Bioactive Compounds: Effects of the Extract on Corn Oil Stability. Food Res. Int..

[189] Cabas B.M., Icier F. (2021). Ohmic Heating–Assisted Extraction of Natural Color Matters from Red Beetroot. Food Bioprocess Technol..

[190] Nikoomanesh N., Zandi M., Ganjloo A. (2024). Development of Ohmic-Assisted Green Extraction Technique with Ultrasonic and Microwave Pretreatments: Burdock (*Arctium lappa* L.) Root Extract. Chem. Eng. Process.-Process Intensif..

[191] Javed T., Oluwole-ojo O., Zhang H., Akmal M., Breikin T., O’Brien A. (2024). System Design, Modelling, Energy Analysis, and Industrial Applications of Ohmic Heating Technology. Food Bioprocess Technol..

[192] Tobiszewski M., Zabrocka W., Bystrzanowska M. (2019). Diethyl Carbonate as a Green Extraction Solvent for Chlorophenol Determination with Dispersive Liquid–Liquid Microextraction. Anal. Methods.

[193] Morón-Ortiz Á., Mapelli-Brahm P., Meléndez-Martínez A.J. (2024). Sustainable Green Extraction of Carotenoid Pigments: Innovative Technologies and Bio-Based Solvents. Antioxidants.

[194] Bosiljkov T., Dujmić F., Cvjetko Bubalo M., Hribar J., Vidrih R., Brnčić M., Zlatic E., Radojčić Redovniković I., Jokić S. (2017). Natural Deep Eutectic Solvents and Ultrasound-Assisted Extraction: Green Approaches for Extraction of Wine Lees Anthocyanins. Food Bioprod. Process..

[195] Zhang H., Zhao W., Liu L., Wen W., Jing X., Wang X. (2023). Switchable Deep Eutectic Solvents for Sustainable Extraction of β-Carotene from Millet. Microchem. J..

[196] Domonkos M., Tichá P., Trejbal J., Demo P. (2021). Applications of Cold Atmospheric Pressure Plasma Technology in Medicine, Agriculture and Food Industry. Appl. Sci..

[197] Ollegott K., Wirth P., Oberste-Beulmann C., Awakowicz P., Muhler M. (2020). Fundamental Properties and Applications of Dielectric Barrier Discharges in Plasma-Catalytic Processes at Atmospheric Pressure. Chem. Ing. Tech..

[198] Chizoba Ekezie F.-G., Sun D.-W., Cheng J.-H. (2017). A Review on Recent Advances in Cold Plasma Technology for the Food Industry: Current Applications and Future Trends. Trends Food Sci. Technol..

[199] Loukri A., Kissas T., Kyriakoudi A., Zymvrakaki E., Stratakos A.C., Mourtzinos I. (2024). Coupling of Cold Atmospheric Plasma Treatment with Ultrasound-Assisted Extraction for Enhanced Recovery of Bioactive Compounds from Cornelian Cherry Pomace. Food Chem..

[200] Bao Y., Reddivari L., Huang J.-Y. (2020). Development of Cold Plasma Pretreatment for Improving Phenolics Extractability from Tomato Pomace. Innov. Food Sci. Emerg. Technol..

[201] Murakonda S., Dwivedi M. (2022). Combined Use of Pulse Ultrasound–Assisted Extraction with Atmospheric Cold Plasma: Extraction and Characterization of Bioactive Compounds from Wood Apple Shell (*Limonia acidissima*). Biomass Conv. Bioref..

[202] Wang X.Y., Han Z.T., Dong Z.Y., Zhang T.H., Duan J.W., Ai L., Xu Y.Y. (2024). Atmospheric-Pressure Cold Plasma-Assisted Enzymatic Extraction of High-Temperature Soybean Meal Proteins and Effects on Protein Structural and Functional Properties. Innov. Food Sci. Emerg. Technol..

[203] Khumsupan D., Lin S.-P., Hsieh C.-W., Santoso S.P., Chou Y.-J., Hsieh K.-C., Lin H.-W., Ting Y., Cheng K.-C. (2023). Current and Potential Applications of Atmospheric Cold Plasma in the Food Industry. Molecules.

[204] ‘Aqilah N.M.N., Rovina K., Felicia W.X.L., Vonnie J.M. (2023). A Review on the Potential Bioactive Components in Fruits and Vegetable Wastes as Value-Added Products in the Food Industry. Molecules.

[205] Sharma P., Osama K., Gaur V.K., Farooqui A., Varjani S., Younis K. (2023). Sustainable Utilization of *Citrus limetta* Peel for Obtaining Pectin and Its Application in Cookies as a Fat Replacer. J. Food Sci. Technol..

[206] Yashini M., Sahana S., Hemanth S.D., Sunil C.K. (2021). Partially Defatted Tomato Seed Flour as a Fat Replacer: Effect on Physicochemical and Sensory Characteristics of Millet-Based Cookies. J. Food Sci. Technol..

[207] Khanpit V.V., Tajane S.P., Mandavgane S.A. (2021). Dietary Fibers from Fruit and Vegetable Waste: Methods of Extraction and Processes of Value Addition. Biomass Conv. Bioref..

[208] Kumar K.A., Sharma G.K., Khan M.A., Govindaraj T., Semwal A.D. (2015). Development of Multigrain Premixes—Its Effect on Rheological, Textural and Micro-Structural Characteristics of Dough and Quality of Biscuits. J. Food Sci. Technol..

[209] Pratelli G., Carlisi D., D’Anneo A., Maggio A., Emanuele S., Palumbo Piccionello A., Giuliano M., De Blasio A., Calvaruso G., Lauricella M. (2022). Bio-Waste Products of *Mangifera indica* L. Reduce Adipogenesis and Exert Antioxidant Effects on 3T3-L1 Cells. Antioxidants.

[210] Pérez-Jiménez J., Saura-Calixto F. (2018). Fruit Peels as Sources of Non-Extractable Polyphenols or Macromolecular Antioxidants: Analysis and Nutritional Implications. Food Res. Int..

[211] Colombo R., Pellicorio V., Barberis M., Frosi I., Papetti A. (2024). Recent Advances in the Valorization of Seed Wastes as Source of Bioactive Peptides with Multifunctional Properties. Trends Food Sci. Technol..

[212] Hordyjewicz-Baran Z., Wasilewski T., Zarębska M., Seweryn A., Zajszły-Turko E., Stanek-Wandzel N., Chrobak J. (2024). Application of Aggregation Behavior of Nonionic Surfactants to Develop a Smart Detergent for Washing Fruits with Emphasis on Pesticide Residues Removal. J. Surfact. Deterg..

[213] Aganovic K., Hertel C., Vogel R.F., Johne R., Schlüter O., Schwarzenbolz U., Jäger H., Holzhauser T., Bergmair J., Roth A. (2021). Aspects of High Hydrostatic Pressure Food Processing: Perspectives on Technology and Food Safety. Compr. Rev. Food Sci. Food Saf..

[214] Guddi K., Sarkar A. (2024). Optimization of Green Extraction Technologies for Recovering Bioactive Compounds from Ixora Coccinea Waste Flower Biomass: A Comparative Response Surface Methodology and Artificial Neural Network Modeling. Sustain. Chem. Pharm..

[215] Rached R.A., Perra M., Manca M.L., Rajha H.N., Louka N., Maroun R.G., Firoznezhad M., Manconi M. (2024). Exploring the Efficacy and Industrial Potential of Polyphenol Products from Grapes and Their By-Products. Sustain. Chem. Pharm..

